# Characterization of LINE-1 Ribonucleoprotein Particles

**DOI:** 10.1371/journal.pgen.1001150

**Published:** 2010-10-07

**Authors:** Aurélien J. Doucet, Amy E. Hulme, Elodie Sahinovic, Deanna A. Kulpa, John B. Moldovan, Huira C. Kopera, Jyoti N. Athanikar, Manel Hasnaoui, Alain Bucheton, John V. Moran, Nicolas Gilbert

**Affiliations:** 1 Institut de Génétique Humaine, CNRS, UPR 1142, Montpellier, France; 2 Department of Human Genetics, University of Michigan Medical School, Ann Arbor, Michigan, United States of America; 3 Cellular and Molecular Biology Program, University of Michigan Medical School, Ann Arbor, Michigan, United States of America; 4 Department of Internal Medicine, University of Michigan Medical School, Ann Arbor, Michigan, United States of America; 5 Howard Hughes Medical Institute, Chevy Chase, Maryland, United States of America; Stanford University, United States of America

## Abstract

The average human genome contains a small cohort of active L1 retrotransposons that encode two proteins (ORF1p and ORF2p) required for their mobility (*i.e.,* retrotransposition). Prior studies demonstrated that human ORF1p, L1 RNA, and an ORF2p-encoded reverse transcriptase activity are present in ribonucleoprotein (RNP) complexes. However, the inability to physically detect ORF2p from engineered human L1 constructs has remained a technical challenge in the field. Here, we have employed an epitope/RNA tagging strategy with engineered human L1 retrotransposons to identify ORF1p, ORF2p, and L1 RNA in a RNP complex. We next used this system to assess how mutations in ORF1p and/or ORF2p impact RNP formation. Importantly, we demonstrate that mutations in the coiled-coil domain and RNA recognition motif of ORF1p, as well as the cysteine-rich domain of ORF2p, reduce the levels of ORF1p and/or ORF2p in L1 RNPs. Finally, we used this tagging strategy to localize the L1–encoded proteins and L1 RNA to cytoplasmic foci that often were associated with stress granules. Thus, we conclude that a precise interplay among ORF1p, ORF2p, and L1 RNA is critical for L1 RNP assembly, function, and L1 retrotransposition.

## Author Summary

Long Interspersed Element-1 (LINE-1 or L1) sequences are the predominant class of autonomous retrotransposons in the human genome and comprise an astounding 17% of human DNA. Although the majority of L1s are considered to be “dead,” an average human genome contains ∼80–100 active L1s. Active L1s encode two proteins (ORF1p and ORF2p) that are required for mobility (retrotransposition) by a “copy and paste” mechanism termed target-site primed reverse transcription. Prior experiments suggested that ORF1p, ORF2p reverse transcriptase activity, and L1 mRNA associate in ribonucleoprotein (RNP) particles and that RNP formation is a necessary step in L1 retrotransposition. However, the difficulty in detecting ORF2p from engineered human L1s has prevented a thorough understanding of its role in L1 retrotransposition. Here, we have exploited epitope and/or RNA–tagging strategies to detect and characterize a “basal” RNP complex from engineered human L1s. We also expanded on previous studies and characterized how mutations in conserved functional domains of ORF1p and ORF2p can adversely affect L1 RNP formation/function. Finally, our strategy allowed us to detect the L1–encoded proteins and L1 RNA in cytoplasmic foci. Thus, we have developed and employed a system to gain greater understanding of LINE-1 retrotransposition at the molecular level.

## Introduction

Long Interspersed Element-1 (LINE-1 or L1) sequences comprise 17% of human DNA and represent the predominant class of autonomous retrotransposon-derived sequences in the genome [1]. Greater than 99.9% of L1 elements are molecular fossils that are no longer capable of mobilization (*i.e.*, retrotransposition) [1]–[3]. However, the average human genome still harbors a small cohort (approximately 80–100) of retrotransposition-competent L1s (RC-L1s) [4], [5]. A wealth of experimental evidence suggests that ongoing RC-L1 retrotransposition has the potential to impact the genome by a myriad of mechanisms (reviewed in [6]–[8]).

**Figure 1:**
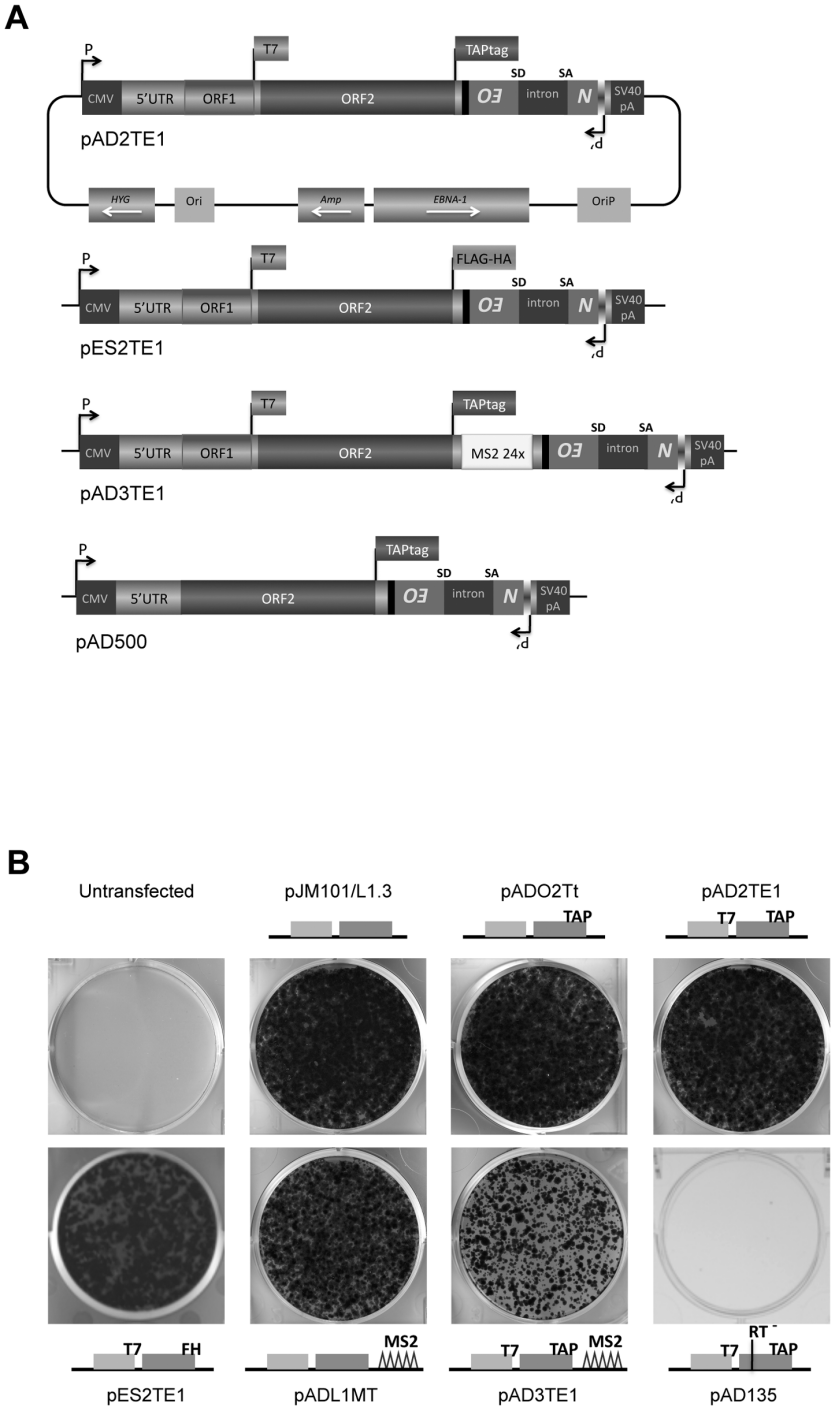
The retrotransposition efficiency of engineered L1s used in this study. *A. A diagram of L1 plasmids used in this study:* Each plasmid is a derivative of pJM101/L1.3 or pDK101 [4], [16]. The constructs were tagged with the *mneoI* retrotransposition indicator cassette [32], [47], and are expressed from the pCEP4 episomal vector (Invitrogen). Labeled rectangles indicate the relative positions of the L1 5′UTR, ORF1p and ORF2p. Labeled flags at the 3′ ends of ORF1 and/or ORF2 are used to denote the epitope tag in the respective constructs. pAD3TE1 also contains 24 copies of a stem-loop sequence that can bind the phage MS2 protein (light rectangle labeled MS2 24x) [65]. pAD500 is a monocistronic ORF2p expression vector that lacks ORF1 as well as the inter-ORF spacer sequence. *B. Representative results of the retrotransposition assay:* L1 retrotransposition efficiency was assayed as described previously [32], [48]. HeLa cells transfected with pJM101/L1.3 serve as a positive control. Untransfected HeLa cells and HeLa cells transfected with an RT mutant (pAD135; D_702_A) serve as negative controls. Cartoons of constructs used in the experiment are indicated in the figure. All constructs contain the *mneoI* retrotransposition indicator cassette.

A human RC-L1 is approximately 6 kb in length; it begins with a ∼910 bp 5′ untranslated region (UTR) that harbors an internal RNA polymerase II promoter [9]–[11], two non-overlapping open reading frames (ORF1 and ORF2), and ends with a 3′ UTR that is followed by either a polyadenylic acid (poly A) or A-rich sequence (Figure 1A) [12], [13]. Genetic and biochemical evidence suggest that the ORF1 and ORF2-encoded proteins (ORF1p and ORF2p, respectively) preferentially associate with their encoding mRNA *in cis* to form a ribonucleoprotein particle (RNP) that probably is an intermediate in the retrotransposition process [14]–[19]. The resultant RNP then gains access to the nucleus, where L1 integration presumably occurs by target-site primed reverse transcription (TPRT) [20]–[23].

Studies conducted with mouse and human RC-L1s have uncovered a number of conserved domains within ORF1p that are important for retrotransposition. The amino acid sequence of the ORF1p amino-terminus is poorly conserved among mammalian L1s, but it is predicted to form a coiled-coil or α-helical domain that is important for ORF1p multimerization [15], [24]–[27]. In human ORF1p, this region contains a putative leucine zipper (LZ) domain that is absent from other mammalian L1s, although a similar motif is present in the L1-like Swimmer element of teleosts [15], [24], [27]–[29]. The coiled-coil domain of ORF1p is followed by a RNA recognition motif (RRM) [30], and experiments in cultured human cells have shown that mutations in conserved residues of the RRM domain (*e.g*., a N_157_A/R_159_A double mutant) adversely affect L1 retrotransposition and the formation of cytoplasmic structures known as ORF1 cytoplasmic foci [31].

The carboxyl-terminus of ORF1p contains amino acid residues that are conserved among mammalian L1s [24], [27], [32]. Biochemical analyses have shown that mouse ORF1p homotrimers bind L1 RNA in a sequence independent manner [33], [34]. Mutations of a conserved di-arginine motif (RR_261–262_ in human L1) can decrease ORF1p RNA binding or mouse ORF1p nucleic acid chaperone activity [33], [35]. Similarly, studies using human L1s revealed that alanine mutations in conserved amino acid residues in the carboxyl terminus of ORF1p (RR_261–262_, and YPAKLS_282–287_, respectively) both compromise the ability of ORF1p to localize to RNPs and severely reduce L1 retrotransposition efficiency [16], [32]. Thus, ORF1p is postulated to have critical functions at discrete steps in the retrotransposition pathway.

Biochemical and genetic studies have revealed that human and mouse ORF2 are translated by an unconventional mechanism [36]–[39]. It is hypothesized that as few as one or two molecules of ORF2p are translated per L1 RNA molecule, which could explain why it has been difficult to detect ORF2p produced from engineered L1s in cultured cells [37]. ORF2p contains endonuclease (EN) and reverse transcriptase (RT) activities that are critical for the target-site cleavage and reverse transcription steps of TPRT [22], [23], [32], [40]. ORF2p also contains a conserved cysteine-rich (C) domain near its carboxyl-terminus [27], [41]. Mutations in the C-domain adversely affect L1 retrotransposition [32]; however, the biochemical role of the C-domain in L1 retrotransposition remains poorly understood.

Epitope-tagging systems and enzymatic assays have been developed to facilitate detection of L1 ORF1p and ORF2p RT activity from engineered wild-type and mutant human L1s [16], [17]. However, the inability to reliably and directly detect ORF2p from engineered human L1s in transfected cultured human cells has hindered progress in the field [37], [42]. Here, we have devised an epitope and/or RNA-tagging system to show that ORF1p, ORF2p, and L1 RNA form a ribonucleoprotein complex, which may represent a minimal RNP retrotransposition intermediate. Consistent with previous studies, transient transfection/immunofluorescence-based experiments revealed that the L1-encoded proteins and L1 mRNA often form discrete cytoplasmic foci, and that many of these foci associate with stress granules [31]. Finally, we have extended previous analyses [16], [17] and demonstrate that mutations in conserved functional domains of ORF1p and/or ORF2p adversely affect L1 RNP formation, the reverse transcription of L1 RNA, and L1 cytoplasmic foci formation. Thus, we have developed a system that should allow a greater understanding of the L1 retrotransposition mechanism at the molecular level.

## Results

### A system to detect L1 ORF2p in cultured cells

Previous studies have examined the co-localization of L1 ORF1p and L1 RNA in RNPs derived from cells transfected with epitope-tagged wild-type or mutant human L1 expression constructs [16], [17]. To physically detect L1 ORF2p, we modified existing L1 expression vectors (pJM101/L1.3 or pDK101) to contain either a 530 bp TAP tag or a 72 bp FLAG-HA tag on the carboxyl-terminus of ORF2p (Figure 1A; pAD2TE1 and pES2TE1) [43], [44]. To facilitate the identification of L1 RNA, we also introduced a 1312 bp DNA fragment that contains 24 copies of a stem-loop sequence that can bind the phage MS2 protein into the L1 3′UTR (Figure 1A; pAD3TE1) [45], [46]. As a control, we generated a plasmid that expresses TAP-tagged ORF2p from a monocistronic transcript (Figure 1A; pAD500).

L1 constructs were equipped with a retrotransposition indicator cassette (*mneoI*), subcloned into a pCEP4 episomal expression vector, and were assayed for retrotransposition in cultured human HeLa cells [32], [47], [48]. Inclusion of either the TAP or FLAG-HA epitope tag onto the carboxyl-terminus of ORF2p had little effect on the L1 retrotransposition efficiency when compared to a wild-type control construct lacking the tag (Figure 1B; pADO2Tt, pAD2TE1, and pES2TE1 *vs.* pJM101/L1.3). Similarly, the inclusion of the MS2 stem loop sequences into the L1 3′UTR did not dramatically affect L1 retrotransposition efficiency (Figure 1B; pADL1MT *vs.* pJM101/L1.3), although we did observe an approximate 2.7 fold reduction in L1 retrotransposition efficiency from a construct containing both the protein and MS2 tags (Figure 1B; pAD3TE1 *vs.* pJM101/L1.3). As a negative control, we demonstrated that a construct containing a missense mutation in the putative L1 RT active site (pAD135; D_702_A) was defective for retrotransposition (Figure 1B). Thus, engineering epitope and/or RNA tags into the L1 expression vectors is compatible with retrotransposition in cultured cells.

### Physical detection of ORF2p in HeLa cells

**Figure 2:**
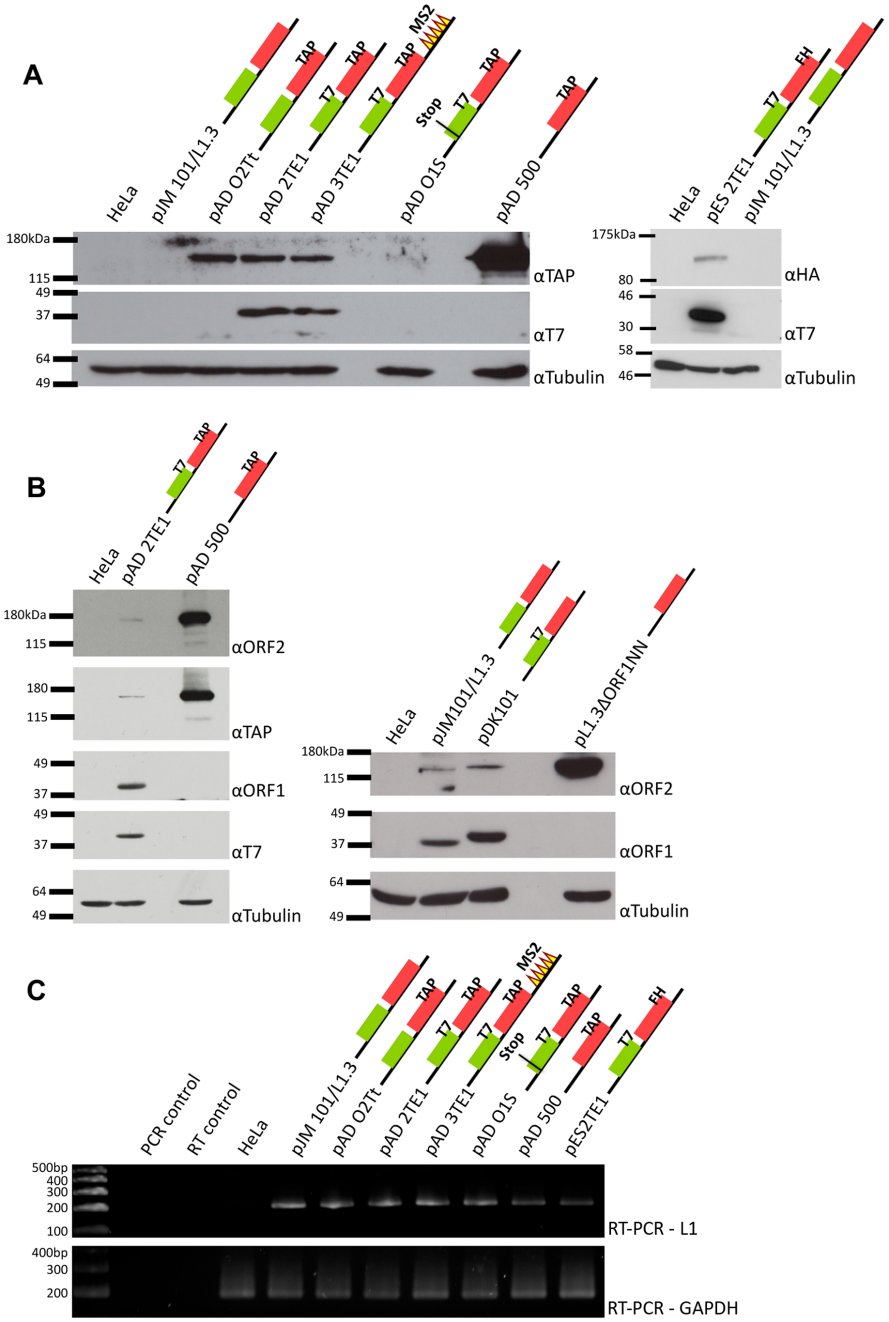
Detection of ORF1p and ORF2p from engineered L1 constructs. *A. Representative results from western blot analyses:* Whole cell lysates derived from untransfected HeLa cells or HeLa cells transfected with the indicated L1 expression constructs were subjected to western blot analyses. Top panels: western blots conducted with anti-TAP antibodies (αTAP; left side) or anti-HA antibodies (αHA; right side) to detect epitope-tagged ORF2p. Middle panels: western blots conducted with anti-T7 antibodies (αT7) to detect epitope-tagged ORF1p. Lower panels: western blots conducted with anti-tubulin antibodies (αTubulin) served as a loading control. Molecular weight standards (Invitrogen, left side, and New England Biolabs, right side) are listed at the left of each series of gels. *B. Protein detection specificity:* Whole cell lysates derived from HeLa cells transfected with the indicated constructs were subjected to western blot analyses using antibodies against either endogenous ORF1p (αORF1) or endogenous ORF2p (αORF2). TAP-tagged ORF2p also was detected using an anti-TAP antibody. T7-tagged ORF1p also was detected with an anti-T7 antibody. Tubulin was detected using an anti-tubulin (αTubulin) antibody and served as a loading control. Molecular weight standards (Invitrogen) are listed at the left of each series of gels. *C. RT-PCR analyses:* RT-PCR reactions using RNAs isolated from whole cell lysates derived from transfected cells revealed that L1 RNA was expressed from each of the constructs. GAPDH mRNA detection was used to assess the quality of the RNA preparations and as a loading control. Reactions without template (PCR control) or reverse transcriptase (RT control) were used as negative controls. DNA size markers (Invitrogen) are indicated at the left of the gel. Colored cartoons of the constructs used in the experiments are indicated next to their respective names. The black lines indicate the 5′ and 3′ UTRs. The green and red boxes indicate ORF1 and ORF2p respectively. When present, epitope tags are indicated. All constructs contain the *mneoI* retrotransposition indicator cassette.

To detect the L1-encoded proteins from the engineered plasmids, we transfected each construct into HeLa cells and selected for cells containing the respective L1 expression vectors by exploiting the hygromycin B selectable marker on the pCEP4 episome (Figure 1A; see Materials and Methods). Consistent with previous studies [16], [17], [49], western blot analyses of whole cell lysates using antibodies directed against the ORF1p T7-epitope tag revealed the presence of a ∼40 kDa protein from constructs containing the tag (Figure 2A, middle panel (αT7); pAD2TE1, pAD3TE1, and pES2TE1), but not from controls lacking the tag (Figure 2A; pJM101/L1.3 and pADO2Tt). We also could detect the ∼40 kDa protein with polyclonal antibodies against endogenous human ORF1p (Figure 2B, αORF1 panels; pAD2TE1, pJM101/L1.3, and pDK101) [50]. Notably, we observed a slight difference in the mobility of T7-tagged and untagged ORF1p (Figure 2B; right panel (αORF1), pJM101/L1.3 *vs.* pDK101), which most likely is due to the additional amino acids imparted by the T7 epitope tag. Controls revealed that ORF1p was not detected from a construct that lacks ORF1 (Figure 2A and 2B; pAD500) or from a construct that contains a premature stop codon in ORF1 (Figure 2A; pADO1S). Qualitative reverse transcriptase-PCR (RT-PCR) experiments further confirmed that L1 RNA was expressed from each of the transfected constructs (Figure 2C; see Oligonucleotides and RT-PCR sections in Materials and Methods for details).

To detect ORF2p expression, we conducted western blot analyses on whole cell lysates derived from transfected cells using antibodies directed against either the TAP or FLAG-HA epitope tag (Figure 2A). A ∼170 kDa protein was detected from L1 constructs containing TAP-tagged ORF2p, but not from an untagged wild-type control (Figure 2A; left panel (αTAP); pADO2Tt, pAD2TE1, pAD3TE1, and pAD500 *vs.* pJM101/L1.3). The ∼170 kDa product corresponds to the predicted size of ORF2p (∼150 kDa) plus the predicted size of the TAP tag (∼19 kDa) [12], [43]. A ∼170 kDa protein also was detected using antibodies against endogenous ORF2p (Figure 2B; left panel (αORF2)) [42]. Similarly, a ∼155 kDa protein was detected from L1 constructs containing FLAG-HA-tagged ORF2p, but not from the untagged wild-type control (Figure 2A; right panel (αHA): pES2TE1 *vs.* pJM101/L1.3). Consistent with previous genetic studies, TAP-tagged ORF2p expression was greatly diminished by introducing a stop codon in ORF1 (Figure 2A; pADO1S) and was most abundant when expressed from an ORF2p monocistronic expression vector (Figure 2A and 2B; pAD500) [37].

### Epitope-tagged ORF2p localizes to ribonucleoprotein particles

**Figure 3:**
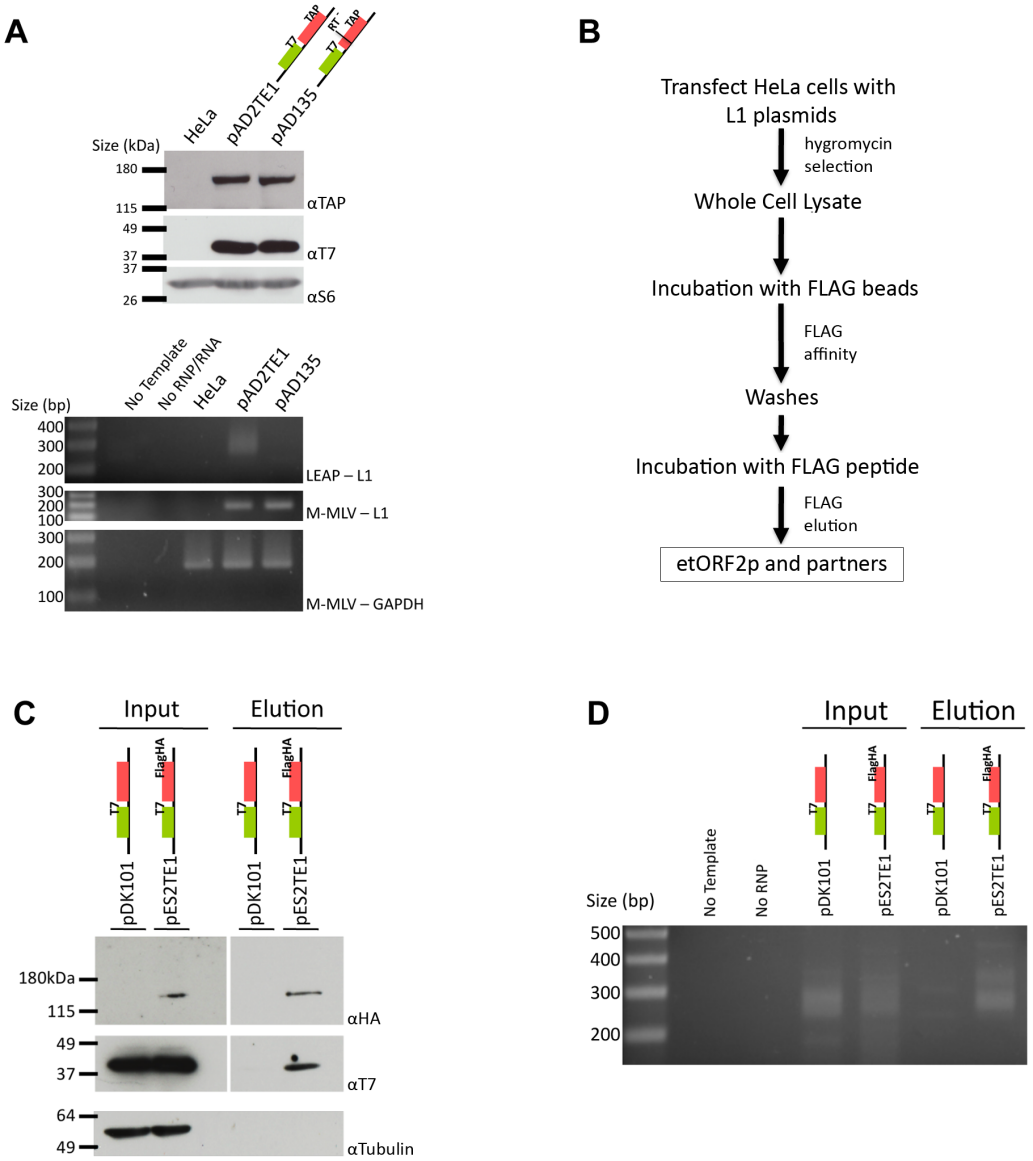
Biochemical identification of a basal L1 RNP complex. *A. L1 RNPs contain ORF1p, ORF2p, L1 RNA, and L1 reverse transcriptase activity:* RNP pellets were obtained from untransfected HeLa cells, or from HeLa cells transfected with wild-type (pAD2TE1) and reverse transcriptase deficient (pAD135) L1 constructs. As in Figure 2, tagged ORF1p and ORF2p were detected using anti-T7 (αT7) and anti-TAP (αTAP) respectively. Ribosomal S6 protein was detected using an anti-S6 (αS6) antibody and was used as an RNP loading control. Reverse transcriptase activity was detected using the LEAP assay as described previously [17]. Reactions without template (No Template) or RNPs (No RNP/RNA) were used as negative controls. Top panel: LEAP reactions (LEAP-L1). Middle panel: L1 RT-PCR reactions conducted with M-MLV reverse transcriptase control for the presence of L1 RNA in RNPs (M-MLV-L1). Bottom panel: GAPDH RT-PCR reactions conducted with M-MLV reverse transcriptase assess RNP RNA quality and serve as a RT-PCR internal control (M-MLV-GAPDH). *B. Flow chart of the L1 RNP immunoprecipitation reaction:* Whole cell extracts were prepared from HeLa cells transfected with either pDK101 or pES2TE1. Immunoprecipitation reactions were conducted by incubating the resultant lysates with agarose beads fused to an anti-FLAG M2 antibody. The elution of ORF2p from the beads was performed by FLAG peptide competition. Western blotting and LEAP assays were performed on aliquots of the whole cell extracts or the elution fractions to detect the L1-encoded proteins and L1-specific reverse transcriptase activity, respectively. *C. Co-immunoprecipitation of ORF1p and ORF2p:* Whole cell extract (input) and immunoprecipitated (elution) products from pDK101 or pES2TE1 transfected cells were subjected to western blotting to identify ORF2p (αHA; top panel), ORF1p (αT7 middle panel) or tubulin (αTubulin, bottom panel). The femto ECL substrate (Pierce) was used to detect ORF1p and ORF2p. *D. A basal L1 RNP complex contains L1 RNA and retains L1 reverse transcriptase specific activity:* LEAP was performed on whole cell extracts (input) or immunoprecipitated (elution) products from pDK101 or pES2TE1 transfected cells. Reactions conducted without template (No Template) or without RNPs (No RNP) were used as negative controls. As in Figure 2, colored cartoons of the constructs are indicated in panels A, C and D. Molecular weight/DNA size markers (Invitrogen) are indicated at the left of the images. All constructs contain the *mneoI* retrotransposition indicator cassette.

To test whether ORF2p localizes to ribonucleoprotein particles (RNPs), we transfected HeLa cells with pAD2TE1, selected for transfected cells, and isolated RNPs by ultracentrifugation (see Materials and Methods) [16], [17]. Western blotting revealed that ORF1p and ORF2p were readily detected in the RNP fraction (Figure 3A, top panel).

We next used the L1 Element Amplification Protocol (LEAP) assay to determine whether the RNP preparations contained an L1-specific reverse transcriptase activity [17]. Consistent with previous studies, a diffuse set of LEAP products that ranged in size from ∼220 to ∼400 bp was detected in pAD2TE1-derived RNPs, but not from pAD135-derived (D_702_A; RT mutant) RNPs (Figure 3A, lower panel). Cloning and sequencing of the pAD2TE1-derived LEAP products confirmed that L1 reverse transcription generally initiated at variable sites within the L1 poly (A) tail, which accounts for variably-sized LEAP products (data not shown [17]).

### Epitope-tagged ORF2p form a complex with ORF1p and their encoding RNA

To further verify that ORF1p, ORF2p, and L1 mRNA form an RNP, HeLa cells were transfected with either pES2TE1 or pDK101. Whole cell extracts then were subjected to immunoprecipitation using an anti-FLAG M2 antibody fused to agarose beads (Figure 3B). Incubation of the beads with a FLAG peptide followed by western blot analysis revealed an enrichment of ORF1p and ORF2p in the pES2TE1, but not in the pDK101 immunoprecipitated reactions (Figure 3C). We sometimes detected a faint band of ∼40 kDa in the pDK101 immunoprecipitated reactions upon longer film exposures, suggesting that some T7-tagged ORF1p may bind non-specifically to the anti-FLAG M2 agarose beads (data not shown). However, subsequent experiments/product characterization determined that the pES2TE1 immunoprecipitated fraction contained LEAP activity, whereas the pDK101 immunoprecipitated fraction lacked a readily detectable LEAP activity (Figure 3D).

Interestingly, we consistently observed less ORF1p associated with RNPs in immunoprecipitation experiments when compared to experiments conducted with whole cell lysates or crude RNPs (Figure 2A and Figure 3A). These data suggest either that ORF1p is less tightly associated with L1 mRNA than ORF2p in RNPs (which is consistent with previous observations [17]) and/or that a fraction of ORF1p is dissociated from L1 RNA during the immunoprecipitation process. Regardless, whereas previous studies showed that ORF1p, ORF2p RT activity, and L1 RNA co-localize to RNPs [16], [17], we were able to demonstrate the physical association of these components in immunoprecipitation experiments.

### Mutations in both ORF1p and ORF2p affect L1 RNP formation and/or function

**Figure 4:**
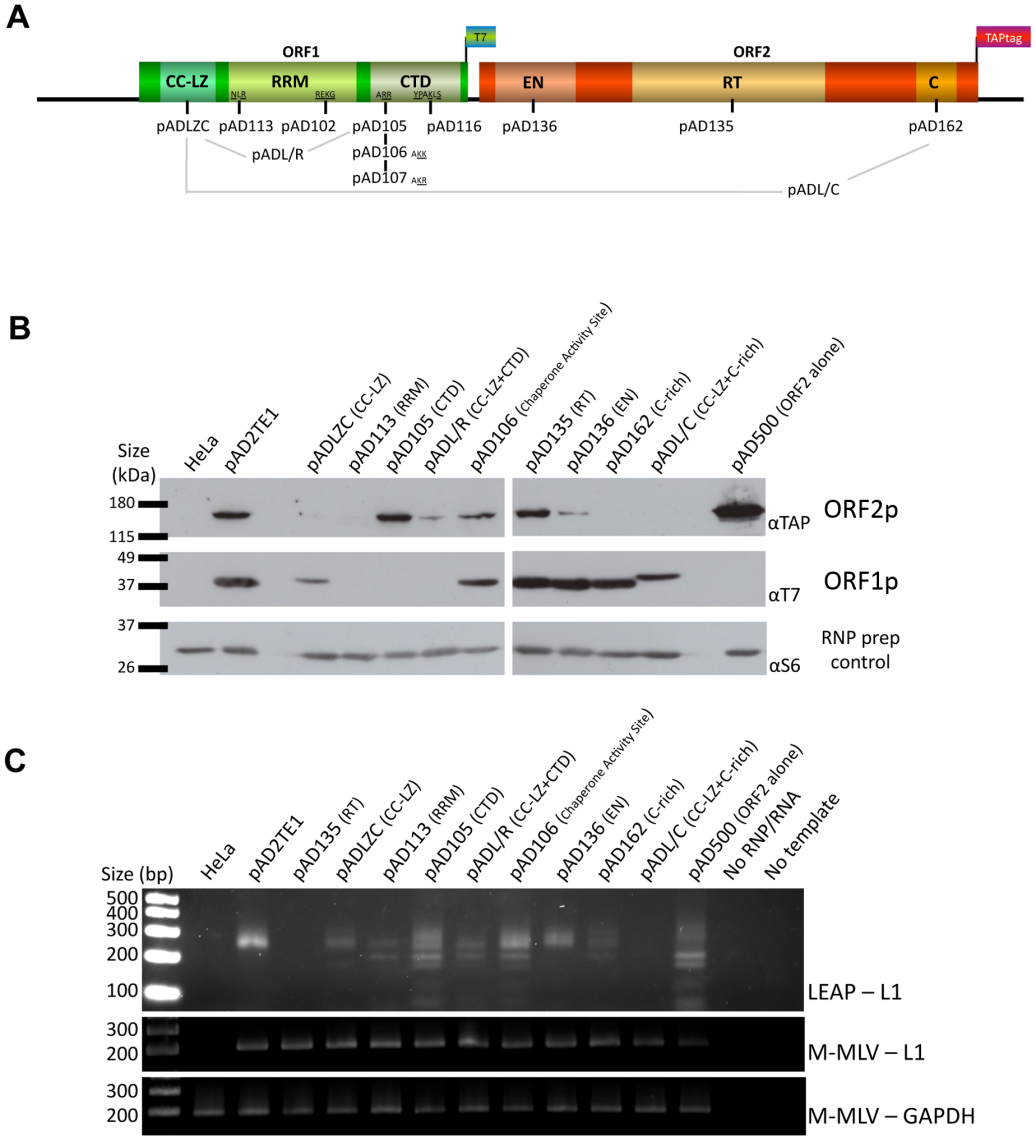
TAP tagged ORF2p and RT activity detection in RNP preparation. *A. Schematic representation of the amino acid mutation positions in L1 sequence:* The names of plasmids containing L1s with mutations in the ORF1p coiled-coil domain (CC-LZ), the ORF1p RNA recognition motif (RRM), and the ORF1p carboxyl-terminal (CTD) domain are indicated below the schematic. The names of plasmids containing mutations in the ORF2p endonuclease domain (EN), reverse transcriptase domain (RT) or cysteine-rich domain (C) also are shown. pADL/R is a double mutant that contains a putative leucine zipper mutation and a carboxyl-terminal domain mutation in ORF1p. pADL/C is a double mutant that contains a putative leucine zipper mutation in ORF1p and a C-domain mutation in ORF2p. The flags indicate the epitope tag present on ORF1 and ORF2. *B. Detection of ORF1p and ORF2p from mutant L1 constructs:* RNPs from HeLa cells transfected with a RC-L1 (pAD2TE1) or the indicated mutant L1 constructs (see Figure 4A) were analyzed by western blotting [16]. Tagged L1 proteins were detected as in Figure 3; ORF2p (top panel), ORF1p (middle panel). Ribosomal S6 protein detection was used as a loading control (bottom panel). Molecular weight markers (Invitrogen) are indicated at the left of the image. *C. L1 RT activity of RNP fractions detected by LEAP:* An aliquot from each of the indicated RNP preparations noted above was used to perform LEAP assays (see Figure 3) [17]. RNPs from pAD2TE1 served as a positive control. RNPs from untransfected HeLa cells or pAD135 (D_702_A; RT mutant) transfected cells served as negative controls. Reactions without RNPs (No RNP/RNA) or template (No Template) also were used as negative controls. Top panel: LEAP reactions (LEAP-L1). Middle panel: L1 RT-PCR reactions conducted with M-MLV reverse transcriptase control for the presence of L1 RNA in the RNP fractions (M-MLV-L1). Bottom panel: GAPDH RT-PCR reactions conducted with M-MLV reverse transcriptase assess RNP RNA quality and serve as a RT-PCR internal control (M-MLV-GAPDH). DNA size markers (Invitrogen) are indicated at the left of the image. All constructs in panel B and C contain the *mneoI* retrotransposition indicator cassette.

Previous studies identified activities associated with ORF1p and ORF2p that are critical for L1 retrotransposition [22], [30], [32], [35]. Here, we expanded on these analyses to determine whether mutations in the L1-encoded proteins affect their ability to localize to RNPs and/or impact L1 reverse transcriptase activity in the LEAP assay. We first tested mutants in the following functional domains of ORF1p: 1) the putative leucine zipper domain (pADLZC; L_93,100,107,114_V); 2) the RNA-recognition motif (pAD113; NLR_157–159_ALA); 3) the carboxyl-terminal nucleic acid binding domain (pAD105; RR_261–262_AA); 4) an ORF1p mutation that affects mouse nucleic acid chaperone activity (pAD106; RR_261–262_KK); and 5) a double mutant in the putative leucine zipper domain and carboxyl-terminal nucleic acid binding domain (pADL/R; L_93,100,107,114_V/RR_261–262_AA) (Figure 4A; see Materials and Methods) [16], [30]–[32], [35]. Each of these mutations, including the LZC mutation (pADLZC; L_93,100,107,114_V), severely compromise L1 retrotransposition efficiency in HeLa cells (Figure S1A, S1B). The LZC mutant data are in agreement with a published report, which demonstrated a L_93/100/114_A triple mutation inactivates L1 retrotransposition [31].

Multiple independent RNP preparations derived from cells transfected with each of the respective mutants were analyzed by western blotting to examine the presence and abundance of both ORF1p and ORF2p (Figure 4B). LEAP assays then were used to determine whether those RNPs contained an L1-specific reverse transcriptase activity (Figure 4C). Once again, control MLV RT-PCR-based experiments, using the same oligonucleotide primers employed in the LEAP assay, indicated that L1 RNA was present at roughly comparable levels in the RNP fraction of HeLa cells transfected with the mutant constructs (Figure 4C).

Consistent with previous data [16], a mutation in the ORF1p carboxyl-terminal domain (pAD105; RR_261–262_AA) led to a severe reduction in the ability of ORF1p, but not ORF2p, to localize to the RNP fraction (Figure 4B). RNPs derived from pAD105-transfected cells had a readily detectable LEAP activity, although the constellation of LEAP products differed from those in the wild-type control, pAD2TE1, because they frequently initiated reverse transcription from within the 3′ end of the L1 mRNA (Figure 4B and 4C; Figure S2A, S2B, S2C and S2D; pDK105 RR_261–262_AA; data not shown). Similar data also were observed for an L1 containing a mutation in the carboxyl-terminal domain (pDK116; YPAKLS_282–287_AAAALA) as well as for pAD500, a TAP-tagged ORF2p construct that lacks ORF1 (Figure 4B and Figure S2A and S2B). These findings support the hypothesis that ORF2p can preferentially associate with its encoding RNA independent of ORF1p binding and that the resultant RNPs retain LEAP activity [17]. Indeed, the constellation of LEAP products observed in the RR_261–262_AA, YPAKLS_282–287_AAAALA, and pAD500 mutants support our previous hypothesis that ORF1p binding to L1 mRNA possibly may restrict hybridization of the LEAP primer to the L1 poly (A) tail [17].

Mutations that affect mouse nucleic acid chaperone activity (pAD106; RR_261–262_KK) had little effect on the ability of ORF1p and ORF2p to localize to RNPs or on LEAP activity (Figure 4B; [16], [35]). We occasionally observed a greater abundance of the lower molecular weight LEAP products, when compared to our wild-type control, pAD2TE1 (Figure 4C). Indeed, closer inspection consistently revealed slightly higher levels of the major LEAP products (∼220 to ∼400 bp) and a slightly lower level of the shorter LEAP products from the RR_261–262_KK mutant (pAD106 and pDK106; Figure 4C, Figure S2B and S2C) when compared to LEAP products derived from the RR_261–262_AA (pAD105 and pDK105; Figure 4C, Figure S2B and S2C) and YPAKLS_282–287_AAAALA mutants (pDK116; Figure S2B). Thus, although the L1 RT activity detected in the LEAP assay does not appear to require ORF1p, it is clear that specific mutations in ORF1p can affect the constellation of products observed in these assays.

Mutations in the putative ORF1p leucine zipper-binding domain (pADLZC; L_93,100,107,114_V) reduced ORF1p and ORF2p localization in the RNP fraction and consistently exhibited lower qualitative levels of LEAP activity when compared to the wild-type control, pAD2TE1 (Figure 4B and 4C). Indeed, quantitative LEAP experiments conducted with pLZC-derived RNPs (a L_93,100,107,114_V mutant that lacks an epitope tag on ORF2p) revealed a five to seven-fold reduction in LEAP activity when compared to a corresponding wild-type control (pDK101; Figure S2E). Subsequent data from LEAP experiments designed to detect variable length L1 cDNA products further suggest that the LZC mutation adversely affects early steps in the reverse transcription of L1 RNA and does not appear to affect L1 RT elongation (Figure S2F).

The putative leucine zipper domain-carboxyl terminal domain double mutant (ADL/R; L_93,100,107,114_V/RR_261–262_AA) shared biochemical characteristics of each single mutant. Similar to pAD105; RR_261–262_AA, ORF1p levels were severely reduced in pADL/R-derived RNPs. However, similar to the putative leucine zipper domain (pADLZC; L_93,100,107,114_V) mutant, ORF2p levels, as well as LEAP activity, were reduced in pADL/R-derived RNPs when compared to the wild-type control, pAD2TE1. Moreover, the LEAP product profile in the double mutant resembled that in the pAD105 mutant (Figure 4B and 4C; Figure S2). Thus, the above data suggest that the LZC mutant adversely affects the accumulation and/or stability of L1 RNPs and that the reduction of ORF2p in RNPs likely contributes to the observed decrease in LEAP activity.

Mutations in the ORF1p RRM domain (pAD113; NLR_157–159_ALA) also led to a severe reduction in the ability of ORF1p and ORF2p to localize to the RNP fraction of transfected cells (Figure 4B). Indeed, ORF2p only was observed upon over-exposure of the resultant western blots (data not shown). The reduced level of ORF2p in pAD113-derived RNPs also correlated with a decrease in LEAP activity when compared to the pAD2TE1 wild-type control (Figure 4B and 4C). Notably, it is unlikely that the NLR_157–159_ALA mutation dramatically affects ORF2 translation because we can detect ORF2p from this mutant by immunofluorescence (see below). Moreover, preliminary data (n = 4 independent experiments) indicates that the NLR_157–159_ALA mutant can serve as a “driver” in a genetic-based *trans*-complementation assay to mobilize a reporter gene (*ORF1mneoI*; [19]) at roughly 60 to 80% the level of the wild-type control, pAD2TE1-NT (Doucet et al., preliminary data). These data are consistent with previous genetic studies, which suggested that ORF1p binding to L1 RNA is not required for ORF2 translation [37]. Moreover, the data suggest that the NLR_157–159_ALA mutations severely compromise the accumulation and/or stability of L1 RNPs (see Discussion).

We next tested mutants in the following functional domains of ORF2p for their effect on L1 RNP formation and L1 reverse transcriptase activity: 1) the L1 endonuclease domain (pAD136; H_230_A); 2) the L1 reverse transcriptase domain (pAD135; D_702_A); 3) the cysteine-rich domain (pAD162; CWWDC_1143–1147_SWWDS) (Figure 4A) [19], [22], [32]. As expected, the L1 RT mutant (pAD135; D_702_A) did not dramatically affect the ability of ORF1p or ORF2p to localize to RNPs, although it did abolish LEAP activity (Figure 4B and 4C) [17]. These data are consistent with previous suppositions that the D_702_A mutant likely blocks the reverse transcription step in TPRT [17], [32], [40].

We repeatedly observed a slight reduction of ORF2p in RNPs derived from the tested endonuclease mutant, and this reduction correlates with a reproducible decrease in LEAP activity (Figure 4A; pAD136; H_230_A). We also observed a severe reduction of ORF2p, as it was only detected upon longer film exposures (data not shown), and a strong decrease of LEAP activity in RNPs derived from the tested cysteine-rich domain mutant (pAD162; CWWDC_1143–1147_SWWDS). Finally, the leucine zipper/C-domain double mutant (pADL/C; L_93,100,107,114_V/CWWDC_1143–1147_SWWDS) displayed both a reduction of ORF1p in RNPs and a concomitant decrease in LEAP activity (Figure 4B and 4C).

As additional controls for the above experiments, we demonstrated that mutant constructs containing a T7-epitope tag on ORF1p, but lacking an ORF2p epitope tag exhibited similar qualitative LEAP activities as the pAD2TE1 mutant based constructs (Figure S2). We also demonstrated that the amount of T7-tagged ORF1p and TAP-tagged ORF2p in whole cell lysates is similar to that in the RNP fraction for each of the pAD2TE1 mutant constructs, and that these proteins were not enriched in insoluble aggregates in the pellet obtained after cell lysis (data not shown). Thus, we conclude that mutations within discrete functional domains of ORF1p and ORF2p have differential effects on L1 RNP formation/function.

### Immunofluorescence detection of the L1–encoded proteins and L1 mRNA

Previous studies have shown that ORF1p often aggregates in cytoplasmic structures termed cytoplasmic foci [31]. Unlike the RNP assays described above (which detect the steady state amount of ORF1p and ORF2p in the RNP fraction of hygromycin resistant cells ∼9 days post-transfection), the L1 cytoplasmic foci formation assays allows the opportunity to visually detect the L1-encoded proteins and/or L1 RNA when over-expressed ∼48 hours post-transfection.

**Figure 5:**
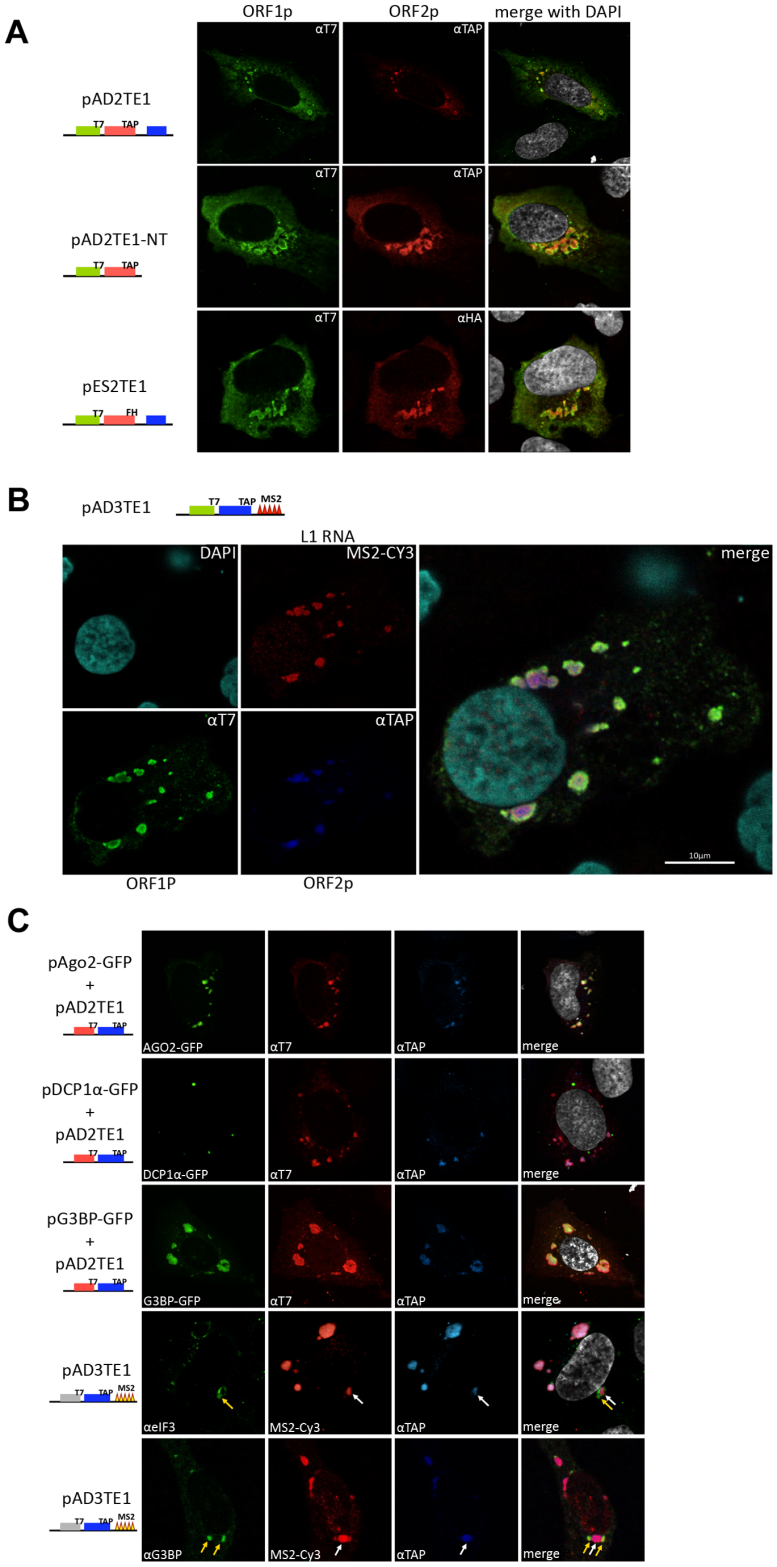
Cellular identification of L1 cytoplasmic foci. *A. Cellular localization of the L1-encoded proteins:* Immunofluorescence was conducted on pAD2TE1 transfected U-2 OS cells 48 hours post-transfection. T7-tagged ORF1p (green; left column) and TAP-tagged ORF2p (red; middle column) staining are shown for representative transfected cells. A merged image is shown in the rightmost column; DAPI (grey) was used to stain nuclear DNA. Cartoons of the constructs are indicated at the left of the micrographs. The blue rectangle in the constructs indicates the *mneoI* cassette. *B. Cellular localization of L1-encoded proteins and RNA:* Immunofluorescence/RNA FISH was conducted on pAD3TE1 transfected U-2 OS cells 48 hours post-transfection. T7-tagged ORF1p (green), TAP-tagged ORF2p (blue), L1 RNA (red), and DAPI (turquoise) staining are indicated in left four micrographs. A merged image is shown in the rightmost panel. The cartoon of pAD3TE1 is shown above the micrographs. *C. L1 cytoplasmic foci are associated with stress granules:* Immunofluorescence/fluorescence microscopy was performed on U-2 OS cells co-transfected with pAD2TE1 and one of the following plasmids: 1) pAgo2-GFP (green staining, top row of images); 2) pDCP1á-GFP (green staining, second row of images); 3) pG3BP-GFP (green staining, third row of images). T7-tagged ORF1p (red), and TAP-tagged ORF2p (blue) also are shown. A merged image is shown in the rightmost panels; DAPI (grey) was used to stain nuclear DNA. Immunofluorescence also was performed on U-2 OS cells transfected with pAD3TE1. Images using antibodies against the endogenous stress granule components eIF3 (áeIF3 (green)) and G3BP (áG3BP (green)) are shown. L1 RNA (red), and ORF2p (blue) also are indicated. Arrows indicate the association of L1 cytoplasmic foci (white) and stress granules (yellow). A merged image is shown in the rightmost columns; DAPI (grey) was used to stain nuclear DNA. All L1 constructs in panel B and C contain the *mneoI* retrotransposition indicator cassette.

To test whether the ORF1p cytoplasmic foci also contain ORF2p and L1 RNA, we conducted immunofluorescence-based localization experiments in a U-2 OS human osteosarcoma cell line that can support the retrotransposition of engineered human L1 constructs (Figure S3A). Initial experiments conducted with pAD2TE1 revealed that ORF1p and ORF2p generally co-localized to discrete cytoplasmic foci 48 hours post-transfection, and that many foci were located near the periphery of the nucleus (Figure 5A). Time course analyses further demonstrated that cytoplasmic foci were apparent in ∼50% of transfected cells as early as 12 hours post-transfection, and that ∼90% of transfected cells displayed cytoplasmic foci 72 hours post-transfection (Figure S3B). ORF1p/ORF2p-containing cytoplasmic foci also were observed in U-2 OS cells transiently transfected with pAD2TE1-NT, which lacks the *mneoI* retrotransposition indicator cassette (Figure 5A) and with a pAD2TE1 derivative lacking the heterologous cytomegalovirus immediate early (CMV) promoter, although foci appeared 24–48 hours later as compared to cells transfected with the wild type control, pAD2TE1 (data not shown). ORF1p and ORF2p co-localization also was observed using an anti-HA antibody to detect ORF2p (Figure 5A; pES2TE1) or antibodies against endogenous ORF1p or ORF2p (Figure S3C; pES2TE1). Qualitatively similar results were obtained when pAD2TE1 was transiently transfected into HeLa or 143Btk cells, which also support L1 retrotransposition [32], [51] (data not shown).

To test whether L1 RNA co-localizes with ORF1p and ORF2p to cytoplasmic foci, we transiently transfected pAD3TE1 into U-2 OS cells. *In situ* hybridization experiments using a fluorescently-labeled probe complementary to the MS2 stem loop structures in the L1 3′UTR revealed the presence of L1 RNA in cytoplasmic foci as well as in nuclei of transfected cells (Figure 5B and Figure S3D). The co-localization of ORF1p, ORF2p, and L1 RNA was confirmed by conducting co-transfection experiments with pAD3TE1 and a plasmid expressing a fluorescently labeled MS2 protein (Figure S3D), and by staining with antibodies against ORF1p and ORF2p (Figure S3C). As above, qualitatively similar results were obtained upon transient transfection of pAD3TE1 into HeLa or HEK293 cells, which also support L1 retrotransposition [32], [51] (data not shown).

To determine whether L1 foci are associated with specific cytoplasmic substructures, we co-transfected U-2 OS cells with pAD2TE1 and plasmids that express GFP fusion proteins that can localize to processing bodies (*i.e.*, P-bodies) and/or stress granules. Consistent with previous analyses, ORF1p and ORF2p associated with an Ago2-GFP fusion protein that localizes both to P-bodies and stress granules (Figure 5C; panel 1) [31], [52]. Refining this analysis revealed that ORF1p and ORF2p co-localized with the stress granule marker G3BP-GFP [53], but did not associate with the P-body marker DCP1α-GFP [54] (Figure 5C; panel 2 and 3). By comparison, experiments conducted with fluorescently labeled antibodies specific for eIF3 and G3BP [53], [55] revealed that stress granules appear to closely associate with the L1 foci (Figure 5C, panel 4 and 5).

Together, the above data demonstrate that ORF1p, ORF2p, and L1 mRNA co-localize to cytoplasmic foci when over-expressed from a variety of engineered L1 episomal expression constructs and that many of these cytoplasmic foci associate with stress granules. However, future experiments are needed to determine whether cytoplasmic foci represent accumulation depots for L1 RNPs or if they play an important role in L1 retrotransposition.

### Mutations in ORF1p and ORF2p adversely affect the formation of L1 cytoplasmic foci

**Figure 6:**
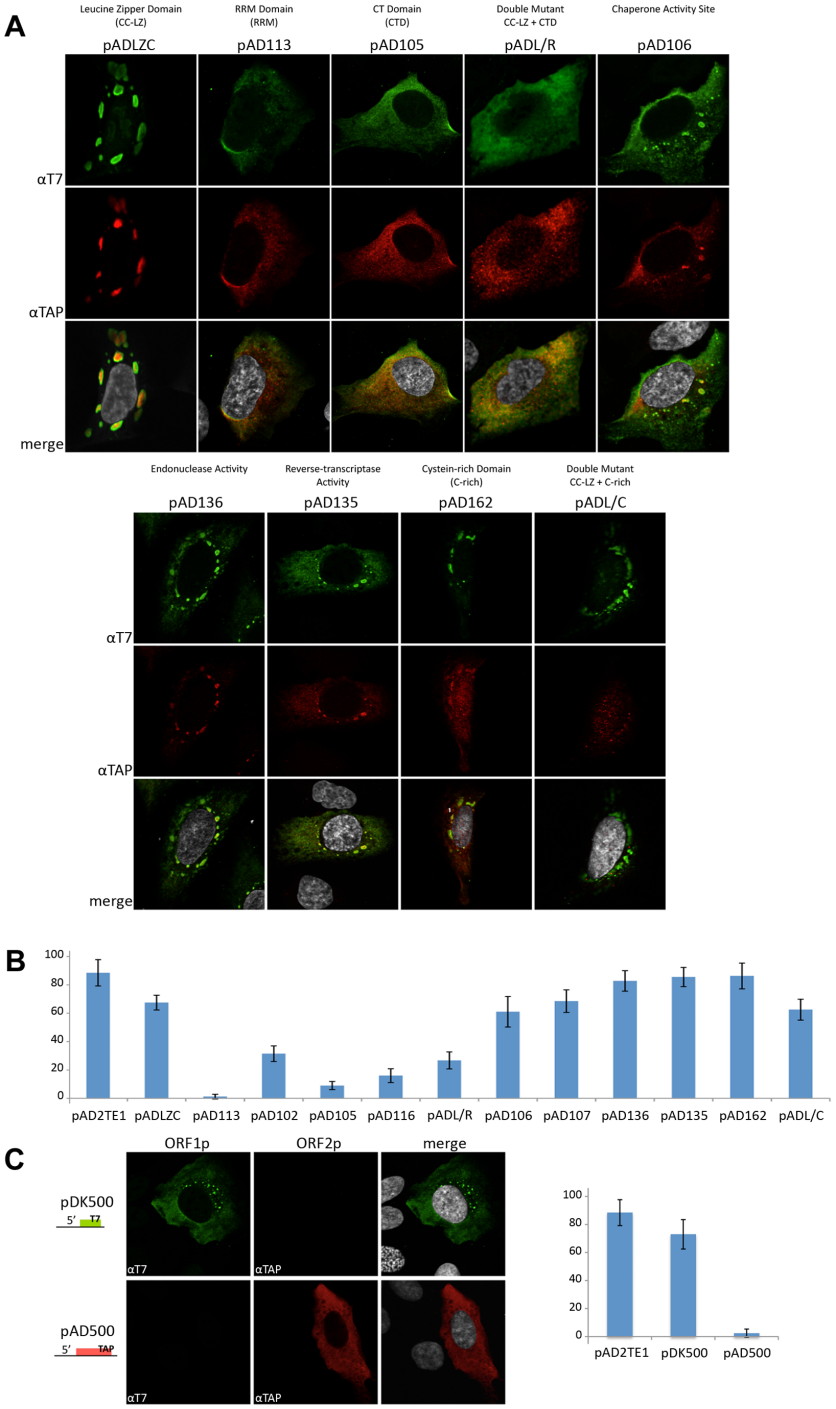
L1 cytoplasmic foci formation requires the nucleic acid binding domain of ORF1p. *A. L1 cytoplasmic foci formation requires the nucleic acid binding domain of ORF1p:* Immunofluorescence was performed on U-2 OS cells transfected with the indicated pAD2TE1-derived mutant plasmids (described in Figure 4A). T7-tagged ORF1p (green; top panels) and TAP-tagged ORF2p (red; middle panels) staining are shown for representative transfected cells. A merged image is shown in the bottom panels; DAPI (grey) was used to stain nuclear DNA. *B. Quantitative analyses of L1 cytoplasmic foci formation:* The number of U-2 OS transfected cells that contains L1 cytoplasmic foci were quantified. The name of the construct used for each transfection is indicated on the X-axis (described in Figure 4A). The percentage of transfected cells displaying L1 cytoplasmic foci is indicated on the Y-axis. pAD2TE1 serves as a positive control. The average of four independent experiments is indicated; error bars  =  standard deviation of the mean. *C. ORF1p is necessary and sufficient for L1 cytoplasmic foci formation:* Immunofluorescence was performed on U-2 OS cells transfected with pDK500 and pAD500. A cartoon of the constructs is shown at the left of the micrographs. T7-tagged ORF1p (green; left column) and TAP-tagged ORF2p (red; middle column) staining are shown for representative transfected cells. A merged image is shown in the rightmost column; DAPI (grey) was used to stain nuclear DNA. The graph indicates the percentage of cells exhibiting L1 cytoplasmic foci. The name of the construct is indicated on the X-axis. The percentage of transfected cells displaying L1 cytoplasmic foci is indicated on the Y-axis. pAD2TE1 serves as a positive control. Four independent analyses of 100 transfected cells were analyzed for each construct. Error bars  =  standard deviation of the mean. All L1 constructs contain the *mneoI* retrotransposition indicator cassette.

We next examined if mutations in the L1-encoded proteins affect L1 cytoplasmic foci formation. Transient transfection of ORF1p mutant expression vectors into U-2 OS cells followed by immunofluorescence staining with anti-T7 and anti-TAP antibodies confirmed that ORF1p and ORF2p are expressed in these cells (Figure 6A). Consistent with previous studies, mutations in the ORF1p RRM domain (pAD113; NLR_157–159_ALA) and carboxyl-terminal RNA binding domain (pAD105; RR_261–262_AA) led to a reduction in the number of L1 cytoplasmic foci (Figure 6A and 6B) [31]. A reduction in the number of L1 cytoplasmic foci also was observed for an RRM domain mutant (pAD102; REKG_235–238_AAAA), an additional carboxyl-terminal domain mutant (pAD116; YPAKLS_282–287_AAAALA), and the putative leucine zipper domain/carboxyl-terminal RNA binding domain double mutant (pADL/R; L_93,100,107,114_V/RR_261–262_AA). By comparison, mutations in the putative ORF1p LZ domain (pADLZC; L_93,100,107,114_V) or mutations that affect the nucleic acid chaperone activity of mouse ORF1p (pAD106; RR_261–262_KK and pAD107; R_261_K) had little effect on L1 cytoplasmic foci formation (Figure 6A and 6B), although we sometimes observed an apparent nucleolar localization of ORF1p in pADLZC transfected cells. None of the ORF2p mutations had a dramatic effect on L1 cytoplasmic foci formation (Figure 6A and 6B), although, we observed a diffuse nuclear localization of TAP-tagged ORF2p in cells transfected with either pAD162 or the putative leucine zipper domain/cysteine-rich domain double mutant (pADL/C; L_93,100,107,114_V/CWWDC_1143–1147_SWWDS) (Figure 6A).

The above data suggest that the ability of ORF1p to bind L1 RNA is critical for L1 cytoplasmic foci formation (Figure 6B). Consistent with this idea, we were able to detect L1 cytoplasmic foci, as well as diffuse ORF1p staining, in U-2 OS cells transiently transfected with a T7-tagged ORF1p expression vector (Figure 6C; pDK500). However, L1 cytoplasmic foci were not detected in U-2 OS cells transiently transfected with a TAP-tagged ORF2p expression vector (Figure 5D; pAD500). Thus, these data, as well as our previously published *trans*-complementation experiments [56], suggest that ORF1p interacts with its encoding RNA *in cis*, and that this association allows L1 cytoplasmic foci formation in the absence of ORF2p.

## Discussion

ORF2p has been notoriously difficult to detect from engineered human L1s in cultured cells. It has been hypothesized that human ORF2p is translated at low levels when compared to ORF1p and/or may be an unstable protein, which might help explain why it has evaded detection [36]–[39], [42]. Previous biochemical studies have identified human ORF2p from vascular endothelial cells *in vivo* [57] and have demonstrated that ORF2p RT activity co-localizes with ORF1p and L1 RNA in cytoplasmic RNPs derived from HeLa cells transfected with wild-type engineered human L1 expression constructs [16], [17]. Here, we have built on these studies and have combined epitope and RNA tagging strategies to physically detect L1 ORF1p, ORF2p and L1 mRNA in cytoplasmic RNPs.

Why our approach allows the ready detection of ORF2p expressed from engineered human L1 constructs requires further study. Experiments conducted with anti-TAP antibodies consistently yielded more robust detection of ORF2p when compared to anti-HA or anti-ORF2p antibodies. Thus, the inclusion of a large carboxyl-terminal tag, such as the TAP-tag, might stabilize ORF2p. However, since engineered L1 constructs containing either the TAP or HA epitope tags on the carboxyl-terminus of ORF2p remain retrotransposition-competent, the strategy described here allows a way to both directly study the expression of ORF1p and ORF2p from a bicistronic transcript and establishes an experimental platform to determine how each protein interacts with L1 RNA. Furthermore, this strategy now allows a comprehensive means to assess how mutations in ORF1p and/or ORF2p affect L1 RNP biogenesis and/or L1 retrotransposition.

**Figure 7:**
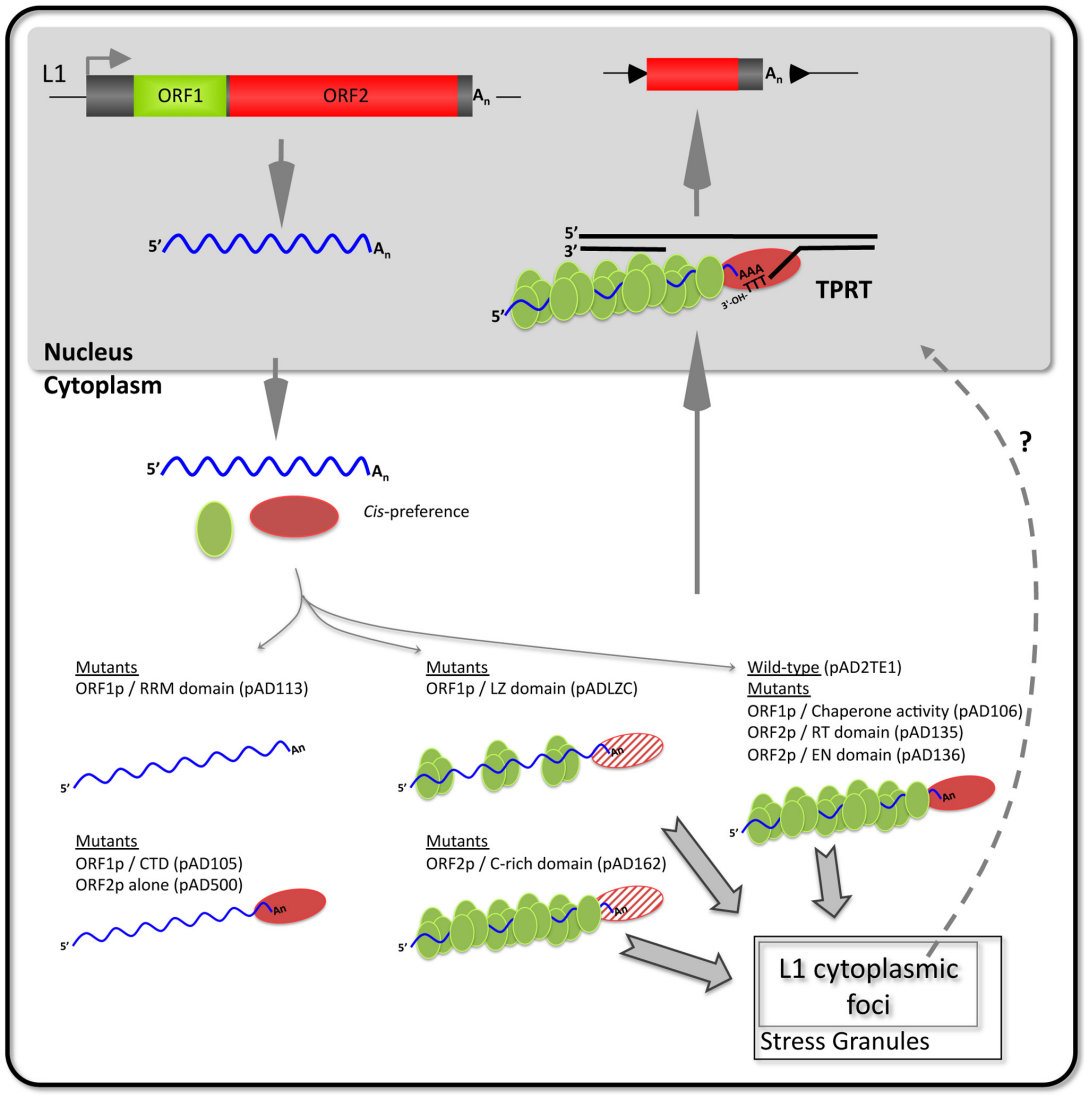
A working model of L1 cytoplasmic RNP formation. A hypothetical model based on our data that builds on previous models of L1 retrotransposition by target-site primed reverse transcription (TPRT; recently reviewed in [6], [8]). ORF1p (green oval), ORF2p (red oval), and L1 RNA (waved blue line) associate with their encoding mRNA via *cis*-preference to form a “basal” retrotransposition complex (right side, pAD2TE1). Mutations in ORF1p and/or ORF2p functional domains have different affects on L1 RNP formation and/or function (thin gray arrows). Mutations in the ORF1p RNA recognition motif (pAD113) disrupt L1 cytoplasmic foci formation and lead to a severe reduction of ORF1p and ORF2p in cytoplasmic RNP complexes (top left side). In some mutants (pAD105 and pAD500) ORF2p can still associate with L1 RNA in the absence of ORF1p RNA binding (bottom left side). Mutations in the putative ORF1p leucine zipper domain (pADLZC) lead to a reduction in ORF1p and ORF2p in RNPs (top center; the reduction in ORF2p is indicated by the striped red oval). Mutations in the ORF2p cysteine-rich domain (pAD162) still allow L1 cytoplasmic foci formation, but adversely affect ORF2p accumulation in RNPs (bottom center). Mutations that disrupt ORF1p nucleic acid chaperone activity (pAD106) or mutations in either the ORF2p endonuclease (pAD136) or reverse transcriptase (pAD135) domains form cytoplasmic RNPs containing ORF1p, ORF2p, and L1 RNA (right side of figure). These mutations probably adversely affect L1 retrotransposition downstream of RNP formation and/or during TPRT. Some RNP complexes localize to L1 cytoplasmic foci and frequently are found in association with stress granules (bold gray arrows). However, whether these foci play a role in L1 retrotransposition remains unknown (indicated by the dotted line and question mark).

Biochemical methods allowed us to assess how mutations in the L1-encoded proteins affect RNP function. For example, in agreement with previous studies, a mutation in the carboxyl-terminal domain of ORF1p (pAD105, RR_261–262_AA) markedly reduced ORF1p levels in RNPs, but did not noticeably affect ORF2p accumulation or LEAP activity (Figure 6A; Figure S3) [16], [17]. Similarly, we could detect ORF2p and LEAP activity in RNPs derived from cells transfected with a construct that lacks ORF1 (Figure 6A and 6B). Thus, we conclude that ORF2p can preferentially associate *in cis* with its encoding transcript to form an RNP independently of ORF1p RNA binding (Figure 7).

Our studies further suggest that an interplay exists between ORF1p, ORF2p and L1 RNA that is critical for proper L1 RNP formation/function (Figure 7). For example, mutations in the putative leucine zipper (pADLZC; L_93,100,107,114_V) or RRM (pAD113; NLR_157–159_ALA) domains led to a reduced amount of ORF2p in the RNP fraction, as well as a decrease in LEAP activity. The L_93,100,107,114_V mutations reside in the N-terminal coiled-coil domain of ORF1p and could potentially alter the structure of the protein. Similarly, the NLR_157–159_ALA mutations reside near coiled-coil domain/RRM junction and structural studies indicate that a hydrogen bond between N_157_ and D_252_ is important for correct folding of the RRM domain [30]. Thus, both of the above mutations may adversely affect the structural integrity of ORF1p, leading to the destabilization of the resultant L1 RNPs. Indeed, such a scenario could potentially account for the reduced levels of ORF2p in RNPs and/or L1 RT activity in these mutants (Figure 7). It is unlikely that the L_93,100,107,114_V, L_93,100,107,114_V/RR_261–262_AA, and NLR_157–159_ALA mutants significantly affect ORF2p translation, since our preliminary data indicate that each mutant can serve as a “driver” in a genetic-based *trans*-complementation assay (Doucet, Hulme *et al*., preliminary data).

As expected, a mutation in the endonuclease domain of ORF2p (pAD136; H_230_A) had no discernable affect on the ability of ORF1p to accumulate in RNPs when compared to a wild-type control construct. However, this mutation consistently led to a slightly reduced amount of ORF2p in RNPs, which correlated with a lower LEAP activity [17]. These findings could potentially explain why the H_230_A mutant consistently exhibited lower levels of endonuclease-independent L1 retrotransposition in Chinese Hamster Ovary cells that are deficient in the non-homologous end-joining pathway of DNA repair when compared to a D_205_A endonuclease domain mutation [58].

Mutations in the cysteine-rich domain (pAD162; CWWDC_1143–1147_SWWDS) did not have a major effect on the ability of ORF1p to accumulate in the RNP fraction. However, these mutations led to a reduced amount of ORF2p in the RNP fraction and a concomitant decrease in LEAP activity when compared to a wild-type control construct. How mutations in the C-domain affect ORF2p accumulation in RNPs requires further study; however, it is possible that these mutations alter the ability of ORF2p to interact with L1 RNA and/or host factors that are important for the biogenesis of L1 RNPs (Figure 7).

A second assay allowed us to determine how mutations in the L1-encoded proteins affect L1 protein expression and cytoplasmic foci formation shortly after transfection. First, we observed that ORF1p and ORF2p can be detected when transiently expressed from the wild-type and mutant L1 constructs used in the study. We next measured the ability of these proteins to form cytoplasmic foci. Consistent with previous studies, retrotransposition-defective L1s containing mutations in either the RRM (pAD113; NLR_157–159_ALA, pAD102; REKG_235–238_AAAA) or carboxyl-terminal domain of ORF1p (pAD105, RR_261–262_AA; pAD116, YPAKLS_282–287_AAAALA) reduced L1 cytoplasmic foci formation [31], [42], [59]. In contrast, mutations in the putative leucine zipper domain (pADLZC; L_93,100,107,114_V) or mutations analogous to those that adversely affect the nucleic acid chaperone activity of mouse ORF1p (pAD106; RR_261–262_KK), which are not predicted to inhibit L1 RNA binding, or mutations in ORF2p had little effect on L1 cytoplasmic foci formation [16], [35]. Thus, unlike our biochemical assays, the L1 cytoplasmic foci formation assay does not allow us to readily assess ORF2p function. Instead, it provides a valuable tool to screen for ORF1p mutations that affect RNA binding or perhaps protein stability (Figure 7).

Consistent with previous studies, we found that L1 cytoplasmic foci are in close association with proteins that are components of stress granules (Figure 7) [31], [59]. Interestingly, recent studies have shown an important role for another cytoplasmic structure (P-bodies) for Ty3 and Ty1 retrotransposition in yeast [60]–[62]. Whether L1 cytoplasmic foci play an important role in L1 retrotransposition awaits further experimentation.

In sum, we have developed a powerful system to physically detect the proteins and RNA encoded by both retrotransposition-competent and mutant L1 constructs in RNP complexes, which now augments previous studies that were based on inferring the presence of ORF2p from its enzymatic activity. It is noteworthy that RNPs derived from the RC-L1s characterized in this study exhibit the biochemical properties predicted of a “basal” L1 retrotransposition intermediate. Thus, we speculate that at least some of the L1 cytoplasmic foci identified here could serve as *bona fide* L1 retrotransposition intermediates. Finally, we predict that the use of the L1 expression constructs developed here will allow a powerful means to identify host factors that play a role in L1 retrotransposition and predict that adaptations of this system will prove useful in identifying RNPs encoded by other non-LTR retrotransposons.

## Materials and Methods

### Oligonucleotides

Sequences of the oligonucleotides used in this study that have been published previously or are available upon request.

3′RACE adapter: 5′- GCGAGCACAGAATTAATACGACTCACTATAGGTTTTTTTTTTTTVN-3′

3′RACE outer: 5′-GCGAGCACAGAATTAATACGACT-3′

GAPDH 3′ end: 5′-GACCCTCACTGCTGGGGAGTCC-3′

Neo Promoter Sens (NPS): 5′-GGTTGCTGACTAATTGAGATGCATGC-3′

Neo8161S: 5′-CACATTCCACAGCTGATCGATACC-3′

L1 3′end: 5′-GGGTTCGAAATCGATAAGCTTGGATCCAGAC-3′

LEAP-86: 5′-CAAACCACAACTAGAATGCAGTG-3′

LEAP-46: 5′-GTGAAATTTGTGATGCTATTGC-3′

### Plasmid constructs

The following plasmids are based on the previously described pJM101/L1.3 and pDK101 constructs [4], [16]. The amino acid and nucleotide numbers indicate the mutation position based on L1.3 accession number L19088 [63]. The constructs were cloned into the pCEP4 expression vector (Invitrogen) and contain the *mneoI* indicator cassette [32], [47] in the L1 3′UTR unless otherwise indicated. PCR followed by subcloning was used to introduce the respective epitope tag sequences onto the 3′ end of ORF2. As a result of this procedure, we deleted a portion of the L1 3′UTR (nts 5818 to 5953). Oligonucleotides used in our cloning strategies are available upon request.

**pADO2Tt** contains a Tandem Affinity Purification epitope tag (TAP tag) [43] on ORF2p and was cloned from the pZome-1-C vector (Euroscarf).

**pAD2TE1** is derived from pDK101 (L1.3) [16] and contains both the T7 *gene 10* epitope tag on the carboxyl-terminus of ORF1p and a TAP tag on the carboxyl-terminus of ORF2p.

**pAD2TE1-Δ2** is derived from pAD2TE1, but lacks CMV promoter and SV40 polyadenylation signal present in the original pCEP4 vector.

**pAD2TE1-NT** is identical to pAD2TE1, but lacks the *mneoI* indicator cassette.

**pES2TE1** is identical to pAD2TE1, but contains a tandem affinity FLAG-HA tag on the carboxyl-terminus ORF2p [44].

**pAD500** is derived from L1.3ΔORF1NN [37], and contains a TAP tag on the carboxyl-terminus of ORF2p.

**pADL1MT** is derived from pJM101/L1.3 and contains 24 repeats of the MS2 stem-loop (MS2 tag) upstream of the *mneoI* indicator cassette in the L1 3′UTR. The MS2 repeats were subcloned from the pTRIP vector [64].

**pAD3TE1** is identical to pAD2TE1, but contains the MS2 tag in the 3′UTR (at the same position as in pADL1MT).

**pADO1S** is identical to pAD2TE1, but contains three stop codons in ORF1. The first two stop codons (R_7_Stop; K_8_Stop) were generated by introducing a thymidine at nucleotide position 928 to create a frameshift mutation and by mutating an A to a T at nucleotide position 930. The third stop codon is from the construct pJM108/L1.3 carrying the mutation S_119_Stop [19], [32].

**pADLZC** is identical to pAD2TE1, but contains four leucine to valine mutations (L_93,100,107,114_V) in the ORF1p putative leucine zipper domain.

**pAD102** is identical to pAD2TE1, but contains the REKG_235–238_AAAA mutations in the ORF1p RRM domain [16], [32].

**pAD105** is identical to pAD2TE1, but contains the RR_261–262_AA mutations in the ORF1p C-terminal domain [16], [19], [32].

**pAD106** is identical to pAD2TE1, but contains the RR_261–262_KK mutations in the ORF1p C-terminal domain [16].

**pAD107** is identical to pAD2TE1, but contains the RR_261–262_KR mutation in the ORF1p C-terminal domain [16].

**pAD113** is identical to pAD2TE1, but contains the NLR_157–159_ALA mutations in the ORF1p RRM domain [31].

**pAD116** is identical to pAD2TE1, but contains the YPAKLS_282–287_AAAALA substitution in the ORF1p C-terminal domain [16], [32].

**pAD135** is identical to pAD2TE1, but contains the D_702_A mutation in the putative ORF2p RT active site [19].

**pAD136** is identical to pAD2TE1, but contains the H_230_A mutation in the ORF2p EN domain [19].

**pAD162** is identical to pAD2TE1, but contains the CWWDC_1143–1147_SWWDS mutations in the ORF2p C-domain [32].

**pADL/R** is identical to pAD2TE1, but contains a putative leucine zipper domain as well as a C-terminal domain mutant (L_93,100,107,114_V; RR_261–262_AA) in ORF1p.

**pADL/C** is identical to pAD2TE1, but contains a putative leucine zipper domain mutation (L_93,100,107,114_V) in ORF1p as well as a C-domain mutation (CWWDC_1143–1147_SWWDS) in ORF2p.

**LZC** is derived from pDK101 and contains four leucine to valine mutations (L_93,100,107,114_V) in the ORF1p putative leucine zipper domain.

**LZ1/2** is derived from pDK101 and contains two leucine to valine mutations (L_93,100_V) in the ORF1p putative leucine zipper domain.

**LZ2/3** is derived from pDK101 and contains two leucine to valine mutations (L_100,107_V) in the ORF1p putative leucine zipper domain.

**LZ3/4** is derived from pDK101 and contains two leucine to valine mutations (L_107,114_V) in the ORF1p putative leucine zipper domain.

**pDK101,** **pDK102,** **pDK105,** **pDK106,** **pDK107,** **pDK108,** **pDK116,** **pDK135,** **and pDK500** were described previously [16].

**pMS2-GFP-nls, pMS2-CFP, and pTRIP** were generous gifts from Edouard Bertrand [64]–[66].

**pAgo2-GFP and pDCP1**α**-GFP** were generous gifts from Gregory Hannon [54].

**pG3BP-GFP** was a generous gift from Jamal Tazi [53].

### Cell culture

Cell lines were maintained in a tissue culture incubator (37°C at a 7% CO_2_ level) in high glucose Dulbecco's modified Eagle medium (DMEM) without pyruvate (GIBCO), supplemented with 10% fetal bovine calf serum and 1X Penicillin-Streptomycin-Glutamine (GIBCO) as described previously [32].

### The L1 retrotransposition assay

The cultured cell retrotransposition assay was conducted as described previously [32], [48]. Briefly, 2×10^4^ HeLa cells/well were plated in 6 well dishes. Within 24 hours, each well was transfected with 1 µg of plasmid DNA (prepared with a Midiprep Plasmid DNA Kit (QIAGEN)) using FuGene-6 transfection reagent (Roche). Three days post-transfection, cells were grown in the presence of G418 (400 µg/mL) to select for retrotransposition events. The media was changed daily. After ∼12 days of selection, the resultant cells were washed with 1X Phosphate-Buffered Saline (PBS), fixed, and stained with crystal violet to visualize colonies. In parallel, HeLa cells were plated in 6 well dishes and transfected with 0.5 µg of the same plasmids and hrGFP (Stratagene). Three days post-transfection cells were subjected to flow cytometry and the transfection efficiency was determined based on the number of GFP positive cells by FACS. In some experiments, 2×10^5^ HeLa cells/well were transfected to monitor L1 retrotransposition.

### Protein expression and western blot analysis

HeLa cells were transfected with a given L1 expression construct in T-25, T-75, or T-175 tissue culture flasks. Whole cell lysates then were prepared after 9 days of hygromycin selection as described previously [16]. The cells were washed in 1X PBS, scraped from plates in 1X PBS, and spun at 3,000 g for 5 minutes at 4°C. One volume of pelleted cells was lysed using two volumes of the following buffer: 1.5 mM KCl, 2.5 mM MgCl_2_, 5 mM Tris-HCl, pH 7.5, 1% deoxycholic acid, 1% Triton X-100, 1X Complete Mini EDTA-free Protease Inhibitor Cocktail (Roche Applied Science). The cells were resuspended by gentle pipetting and incubated on ice for 10 minutes. The lysate was cleared by centrifugation at 3,000 g for 5 minutes at 4°C. Untransfected HeLa cell samples were obtained three days after plating. The Bradford reagent (Bio-Rad) was used to determine the protein concentrations [67]. The same amount of total protein was separated by SDS-PAGE. BenchMark Pre-Stained Protein Ladder (Invitrogen) was used as a molecular weight marker. The proteins were detected by western blot using the following primary antibodies: mouse anti-T7-Tag (Novagen), rabbit anti-TAP (Open Biosystems), rat anti-HA (3F10 clone, Roche), mouse anti-α-tubulin (Sigma), rabbit anti-S6 (Cell Signaling Technology), rabbit anti-ORF1p (a generous gift from Thomas Fanning [50]) and rabbit α-ORF2p-N (a generous gift from John Goodier [42]. Goat anti-mouse, anti-rabbit and anti-rat HRP-conjugated secondary antibodies were purchased from GE/Amersham. Western blots were developed using either the pico or femto ECL substrate (Pierce) according to manufacturer's protocols.

### RNA preparation and RT–PCR analysis

RNA isolation was performed with the RNeasy Kit (QIAGEN) coupled to an on-column DNase treatment (QIAGEN). Whole cell lysates (10–50 µL) were used as starting material. The isolated RNAs were resuspended in Ultrapure distilled water (GIBCO) and quantified using a Nanodrop spectrophotometer (Thermo Scientific). For the LEAP assay controls, RNA was isolated from a 50 µL RNP sample (1.5 mg/mL). RT-PCR was performed on 0.5 µg total RNA, using the 3′RACE adapter primer (0.4 µM) and M-MLV reverse transcriptase (200U) (Promega). The resultant cDNA products then were amplified by PCR using HotStart Pfu Turbo polymerase (Stratagene) with one primer specific to the transfected L1 constructs (L1 3′ end) or GAPDH (GAPDH 3′ end) and the 3′RACE outer primer, as described previously [17]. The PCR cycles were as follows: one cycle at 94°C for 3 minutes, then thirty five cycles of 94°C for 30 seconds, 58°C for 30 seconds and 72°C for 30 seconds. Then, a final extension was performed at 72°C for 10 minutes.

### LEAP assay

The LEAP assay has been described previously [17]. Briefly, HeLa cells were plated at 6×10^6^ cells/flask in T-175 flasks, and transfected within 24 hours with 30 µg plasmid DNA (Midiprep Plasmid DNA Kit (QIAGEN)) using FuGene-6 transfection reagent (Roche). HeLa cells were grown in the presence of hygromycin from days 3 to 9 post-transfection (200 µg/mL) to select for episome-containing cells. HeLa cells grown for three days in the absence of hygromycin served as an untransfected (naïve) control. On day 9, transfected cells and naïve HeLa cells were harvested, lysed, and the cleared whole cell lysates were centrifuged through an 8.5%/17% (w/v) sucrose cushion at 178,000 g for 2 hours. The resultant pellet was resuspended with 100 µL dH_2_O +1X Complete EDTA-free protease inhibitor cocktail (Roche). Bradford reagent (Bio-Rad) was used to determine protein concentration and this RNP sample was diluted to a final concentration of 1.5 mg/mL. An aliquot (1.5 µg) of the RNP sample was added to 49 µL of LEAP assay master mix (50 mM Tris-HCL (pH = 7.5), 50 mM KCl, 5 mM MgCl_2_, 10 mM DTT, 0.4 µM 3′RACE adapter primer, 20U RNasin (Promega), 0.2 mM dNTPs, and 0.05% (v/v) Tween 20) and was incubated at 37°C for 1 hour. LEAP cDNA products (1 µL) were amplified in a standard 50 µL PCR reaction containing 0.4 µM of the 3′RACE outer primer and 0.4 µM of one of the following forward primers: L1 3′ end; Neo promoter sense; Neo8161S; LEAP-86; LEAP-46, using HotStart *Pfu* Turbo polymerase (Stratagene) according to the manufacturer's protocol (see Figure S2). The resultant products were visualized on 2% agarose gels. PCR products were isolated, cloned into the pCR-Blunt vector (Invitrogen), and sequenced to confirm their identity. The diffuse profile of the amplification above 220 bp is explained by initiation of reverse transcription at many places on L1 poly (A). Lower bands, below 200 bp, are due to an internal initiation of reverse transcription 5′ of the poly (A) tail [17].

### Affinity purification

The affinity purification procedure described in Figure 3 was adapted from a published protocol [68]. To prepare the samples, HeLa cells were plated in T-175 flasks and transfected as described in the previous paragraph. Hygromycin selection on days 3 to 9 post-transfection was used to select for cells expressing the respective constructs. Using these conditions, one T-175 flask per plasmid was sufficient to yield enough cellular material (3 mg) for an experiment. Cells were washed, scraped from the flasks in 1X PBS, and centrifuged at 3,000 g for 5 minutes at 4°C. Cells were lysed by repeated pipetting with 3 volumes of IP FLAG buffer (0.1% NP-40, 100 mM KCl, 20 mM Tris-HCl pH 8, 1 mM DTT, 10% Glycerol, 1X complete EDTA free Protease inhibitor (Roche)) and incubated for 15 minutes on ice. The cellular debris was removed by centrifugation at 3,000 g for 5 minutes at 4°C. The protein concentration of the supernatant was quantified by a Bradford assay (Biorad Protein Assay).

For immunoprecipitation reactions, anti-FLAG beads (EZview Red ANTI-FLAG M2 Affinity Gel, Sigma) were equilibrated in 0.1M Glycine (pH 2.2) (5 µL for 100 µL of beads) for 5 minutes at room temperature. After addition of Tris-HCl (pH 8.0) (10 µL for 100 µL of beads), the beads were spun down for 3 minutes at 3000 rpm and then washed 3 times with IP FLAG buffer (mentioned above). For each condition, 3 mg of protein extract (input) was then incubated on a rotating wheel overnight at 4°C with 20 µL of the pre-equilibrated anti-FLAG beads. The next day, the beads were washed 5 times with 1 mL of IP FLAG Buffer for 10 minutes at 4°C. The beads were incubated 1 hour (at 4°C on the wheel) with 200 µL of IP FLAG buffer containing 200 µg/mL of 3X FLAG peptide (Sigma). The elution fraction then was collected and analyzed alongside the corresponding input fraction by western blotting (as described above in the dedicated section). The femto ECL substrate (Pierce) was used in the detection of both T7-tagged ORF1p and FLAG-HA-tagged ORF2p in this experiment. An aliquot (1 µL) of the input and elution samples then were used to perform the LEAP assay (see previous section for detailed protocol).

### Quantitative real-time PCR

Quantitative PCR was performed on LEAP cDNA samples or M-MLV RT-PCR products using the 7300 Real Time PCR system (Applied Biosystems). For analysis, 1 µL of LEAP or M-MLV RT products was added to 19 µL of master mix (1X SYBR Green PCR Master Mix (Applied Biosystems), 500 nM L1 3′ end primer, and 500 nM L1 Reverse primer), and amplified in a standard Q-PCR run of 45 cycles. The average cycle threshold (Ct) value for each experimental or control sample was calculated from three independent reactions within a Q-PCR run. The ‘absolute quantitation by standard curve’ method was used to determine the number of cDNA molecules in each LEAP RNP or RNA sample. A standard curve was generated using dilutions of a L1 LEAP product cloned into a plasmid, and a best fit line (log(molecules) versus average Ct value) for these standards was generated by linear regression. For each wild-type or mutant L1, RNA levels from three independent RNP samples were examined by at least one RT reaction and two Q-PCR runs. The level of LEAP activity in each wild-type or mutant L1 was determined from four independent RNP samples. These RNP samples were characterized by at least one and up to three independent LEAP RT reactions and one or two independent Q-PCR runs. For LEAP activity, the negative control RT- (pDK135) gave a background amplification level of ∼15–30 molecules of cDNA due to the presence of the transfected L1 plasmid in the RNP sample. This RT- background control was included in each Q-PCR run and the background amount of molecules was subtracted from each experimental sample in Figure S2 and when calculating fold changes.

### Fluorescent In Situ Hybridization (FISH) and immunofluorescence

U-2 OS cells were plated at 10^5^ onto sterile glass cover slips in 6 well tissue culture dishes. The following day, cells were transfected using 1 µg of purified plasmid DNA (Midiprep Plasmid DNA Kit, QIAGEN) and 3 µL of FuGene-6 Transfection Reagent (Roche Applied Science). The FISH protocol was adapted from the Robert Singer (Albert Einstein College of Medicine, New York) lab protocol (available at http://www.singerlab.org/protocols) and was modified to allow protein detection by immunofluorescence. Briefly, 48 h post-transfection, cells were washed twice with 1X PBS and fixed with 4% paraformaldehyde in 1X PBS for 10 minutes at room temperature. The fixed cells then were washed 2 additional times with 1X PBS. The fixed cells were permeabilized by treatment with 70% ethanol overnight at 4°C. The following day, cells were rehydrated with 1X saline-sodium citrate (SSC) and 10% formamide for 5 minutes at room temperature. To prepare the hybridization solution, a first mix containing 40 µg of *E.coli* tRNA (Sigma), 1X SSC, 10% formamide, and 7.5 ng of MS2-Cy3 probe (generous gift from Dr. Edouard Bertrand) was boiled for 1 minute at 100°C in order to denaturize the probe. The quantities of probe and tRNA are indicated for hybridization of one slide. A second mix was prepared with 10% dextran sulfate, 2 mM vanadyl-ribonucleoside complex (Sigma), and 0.02% RNase free BSA (Roche Applied Science). After probe denaturation, mixes 1 and 2 were combined to form the final hybridization solution. The re-hydrated cells were hybridized overnight at 37°C in 30 µL of this hybridization solution. Cells were then washed twice for 30 minutes at 37°C with 1X SSC, 10% formamide and 3% BSA and then were incubated with primary antibodies for 1 hour at 37°C. The cells were washed three times with 1X PBS and were incubated with secondary antibodies and 0.2 µg/mL 4′,6′-diamidino-2-phenylindole (DAPI, Molecular Probes) for 30 minutes at 37°C and washed three times with 1X PBS. The primary and secondary antibodies were diluted in 1X PBS and 3% BSA and are as follows: anti-T7 (Novagen), anti-TAP (Open Biosystems), anti-HA (Roche), rabbit anti-ORF1p (a generous gift from Thomas Fanning) [50]) and rabbit α-ORF2p-N (a generous gift from John Goodier) [42], anti-eIF3 (Santa Cruz BioTechnology), anti-G3BP (generous gift from Jamal Tazi), Alexa Fluor 488 anti-mouse and anti-rabbit (Invitrogen), Alexa Fluor 546 anti-mouse and anti-rabbit (Invitrogen), Cy3-conjugated anti-rat (Jackson Immuno Research) and Cy5-conjugated anti-mouse and anti-rabbit (Jackson Immuno Research). Cells were rinsed with water and mounted on slides with Vectashield (Vector Laboratories). Samples were then analyzed with appropriate fluorescent filters on DMRXA Leica microscope and images were captured using a Zeiss LSM510 META confocal microscope.

The above protocol was used for both RNA and protein detection analyses. In experiments where we only sought to detect RNA, the protocol was stopped after hybridization with the MS2-Cy3 probe and subsequent washes. Cover slides were stained with DAPI and mounted on slides as described above. In experiments where we only sought to detect protein, fixed cells were permeabilized by treatment with anhydrous methanol for 1 minute. After three washes with 1X PBS, the cells were incubated with 3% BSA in 1X PBS for 30 minutes. Antibody incubation and DAPI staining were performed as described above. We verified that the protein A domain contained in the TAP tag of ORF2p did not react with the secondary antibodies (data not shown).

### Analysis of L1 cytoplasmic foci

In general, L1 cytoplasmic foci formation was measured 48 hours post-transfection. At least two independent series of slides were analyzed. Each analysis corresponds to 100 transfected cells that were quantified in a blinded manner. Cells in which we were able to distinguish a concentrated cytoplasmic signal from a diffuse cytoplasmic signal using a 63x or 100x objective (equivalent of 1 micrometer of diameter) were considered as L1 cytoplasmic foci.

## Supporting Information

Figure S1 Retrotransposition assays with mutant L1 constructs. A. Retrotransposition assays with mutant L1 constructs: 2×104 HeLa cells were transfected with the indicated constructs. pJM101/L1.3 and pAD2TE1 were used as positive controls. All of the pAD-based constructs contain the ORF1p T7 epitope tag and the ORF2p TAP-tag except for pAD500, which lacks ORF1. pAD135 is an RT mutant (D702A), and serves as a negative control. B. Retrotransposition assay with leucine zipper domain mutants: 2×105 HeLa cells were transfected with the indicated pDK101-derived constructs. T7WT (pDK101) is a wild-type L1 (L1.3) that contains the T7 epitope tag on the carboxyl terminus of ORF1p. pDK101 was modified to create LZ1/2 (L93V, L100V), LZ2/3 (L100V, L107V), LZ3/4 (L107V, L114V), and LZC (L93V, L100V, L107V, L114V). Each of the mutations abolished L1 retrotransposition.
(0.57 MB TIF)

Figure S2 The effect of ORF1p mutations on LEAP activity. A. Results of western blot analyses: RNPs derived from wild-type (pDK101) and the indicated mutant constructs were subjected to western blot analyses with an anti-T7 antibody (αT7). An ∼40 kDa band indicative of epitope-tagged ORF1p is shown. Untransfected HeLa cells served as a negative control. The ribosomal S6 protein was detected using an anti-S6 (αS6) antibody (bottom panel; RNP prep control) and served as a loading control. Molecular weight markers (Invitrogen) are indicated at the left of the gel. B. Results of LEAP assays: Top panel: An aliquot of the above RNPs was used to measure LEAP activity. RNPs derived from wild-type (pDK101) generate strong LEAP products of ∼220–400 bp and served as a positive control. Untransfected HeLa cells and an RT mutant (pDK135; D702A) serve as negative controls. LEAP products generated in ORF1p mutant RNPs are shown. Reactions conducted without template (No Template) or without RNPs (No RNP/No RNA) were used as negative controls. Middle and bottom panels: RT-PCR with M-MLV RT and primers specific to either the transfected L1 constructs or GAPDH confirmed the presence of L1 RNA in RNPs and the integrity of the RNA isolation procedure. DNA size markers (Invitrogen) are indicated at the left of the gel. C. LEAP products derived from the ORF1p RNA binding mutant (RR261-262AA) and putative chaperone mutant (RR261-262KK): Representative LEAP products derived from wild-type (pDK101), an ORF1p RNA binding mutant (pDK105; RR261-262AA), and a putative ORF1p nucleic acid chaperone activity mutant (pDK106; RR261-262KK) are depicted at the top gel. The middle and bottom gels are RT-PCR reactions conducted with M-MLV RT and primers specific to L1 and GAPDH transcripts, respectively. The black arrow on the middle gel indicates the size of the specific L1 cDNA amplification products. Untransfected HeLa cells and a RT mutant (pDK135) served as negative controls. Additional negative controls include reactions conducted without template (No Template) as well as reactions conducted without RNPs or RNA (No RNP/No RNA). DNA size markers (Invitrogen) are indicated at the left of the gel. D. LZC RNPs have decreased reverse transcriptase activity: Top panel: RNPs derived from T7WT (pDK101) generate strong LEAP products of ∼220–400 bp. By comparison, LZC (see Figure S1B) had a less intense band at ∼220–400 bp. LEAP products also were seen in the RR261-262AA (pDK105) mutant and the LZC/RR261-262AA mutants. No product was seen in untransfected HeLa cells or for a L1 containing a RT active site mutation (pDK135; RT-). Middle panels: RT-PCR with M-MLV RT and primers specific to either the transfected L1 constructs or GAPDH confirmed the presence of L1 RNA in RNPs and the integrity of the RNA isolation procedure. No RNP/RT and dH2O served as negative controls. DNA size markers (Invitrogen) are shown at the left side of the gel. Bottom panels: Western blot against the T7 epitope tag detects ORF1p (αT7). Untransfected HeLa cells served as a negative control. Western blot against ribosomal protein S6 was used as a loading control (αS6). Molecular weight markers (Invitrogen) are indicated at the left of the blot. E. Quantitative PCR of LEAP cDNAs from ORF1p LZC and carboxyl-terminal nucleic acid binding domain mutants: Representative results of a Q-PCR run are shown. Standard deviations are indicated on the graph. Q-PCR was performed on four independent RNP preps for T7WT (pDK101), LZC, RR261-262AA (pDK105), and the LZC/RR261-262AA double mutant. Three of four preps showed a 5–7 fold decrease in LZC LEAP activity compared to wild-type. One RNP prep showed a 16–23 fold decrease in LZC LEAP activity compared to wild-type. F. LZC mutation does not appear to affect reverse transcriptase elongation: Top panel: PCR was performed on LEAP cDNAs using different primers pairs. The names of the primers and the approximate size of each product are indicated in the cartoon above the gel. LZC RNPs yield fewer products when compared to T7WT (pDK101) RNPs with all tested primer sets. Bottom panel: RT-PCR with M-MLV RT confirms the presence of L1 RNA in the RNP samples. DNA size markers (Invitrogen) are indicated at the left of the gel. The L1 constructs in all panels contain the mneoI retrotransposition indicator cassette.
(1.42 MB TIF)

Figure S3 L1 retrotransposition and L1 cytoplasmic foci formation in U-2 OS cells. A. L1 retrotransposition assays: 2×104 cells were transfected with the indicated L1 constructs. pJM101/L1.3 and pAD2TE1 were used as positive controls. Untransfected cells and pAD135 (RT mutant (D702A)) serve as negative controls. A cartoon of each L1 is shown above the tissue culture dishes. Green rectangle  =  ORF1; Red rectangle  =  ORF2. The relative positions of the T7 and TAP tags also are indicated. All constructs contain the mneoI retrotransposition indicator cassette. B. Time course analyses of L1 cytoplasmic foci formation: Cells were transfected with pAD2TE1. X-axis  =  time after transfection. Y-axis  =  percentage of transfected cells containing L1 cytoplasmic foci. For each time point, 100 transfected cells were analyzed for the presence of ORF1p and ORF2p in L1 cytoplasmic foci. Error bars  =  standard deviation (n = 3). C. Cytoplasmic localization of L1 proteins and RNA: Top panels: Cells were transfected with pAD3TE1. ORF2p was visualized with an anti-ORF2 antibody (αORF2, green). ORF1p was visualized with an anti-T7 antibody (αT7, blue). L1 RNA was visualized with an MS2-Cy3 FISH probe (red). A merged image is shown in the rightmost column; DAPI (grey) was used to stain nuclear DNA. Middle panels: Cells were transfected with pES2TE1. ORF2p was visualized with an anti-ORF2 antibody (αORF2, green) and an anti-HA antibody (αHA, red). ORF1p was visualized with an anti-T7 antibody (αT7, blue). A merged image is shown in the rightmost column; DAPI (grey) was used to stain nuclear DNA. Bottom panels: Cells were transfected with pES2TE1. ORF1p was visualized with an anti-ORF1 antibody (αORF1, green) and an anti-T7 antibody (αT7, blue). ORF2p was visualized with an anti-HA antibody (red). A merged image is shown in the rightmost column; DAPI (grey) was used to stain nuclear DNA. D. Localization of L1 RNA: Top panels: Cells were transfected with pADL1MT. L1 RNA was visualized with an MS2-Cy3 FISH probe (MS2-Cy3, red). DAPI was used to stain nuclear DNA (gray). A merged image is shown in the rightmost column. Middle panels: Cells were co-transfected with pAD3TE1 and pMS2-GFP-nls. Fluorescence was used to visualize MS2-GFP-nls (green). L1 RNA was visualized with an MS2-Cy3 FISH probe (MS2-Cy3, red). ORF1p was visualized with an anti-T7 antibody (αT7, blue). A merged image is shown in the rightmost column. Bottom panels: Cells were co-transfected with pAD3TE1 and pMS2-CFP. Fluorescence was used to visualize MS2-CFP (green). L1 RNA was visualized with an MS2-Cy3 FISH probe (MS2-Cy3, red). ORF2p was visualized with an anti-TAP antibody (αTAP, blue). A merged image is shown in the rightmost column. The use of MS2 binding protein system confirms the cytoplasmic localization of the L1 RNA observed by FISH as well as the co-localization of L1 RNA with ORF1p and ORF2p. The L1 constructs in all panels contain the mneoI retrotransposition indicator cassette.
(2.01 MB TIF)
