# Supplementary figures and images for: Characterization of LINE-1 Ribonucleoprotein Particles
Source: PLoS Genet. 2010 Oct 7;6(10):e1001150. doi: 10.1371/journal.pgen.1001150 (PMC2951350; doi:10.1371/journal.pgen.1001150)

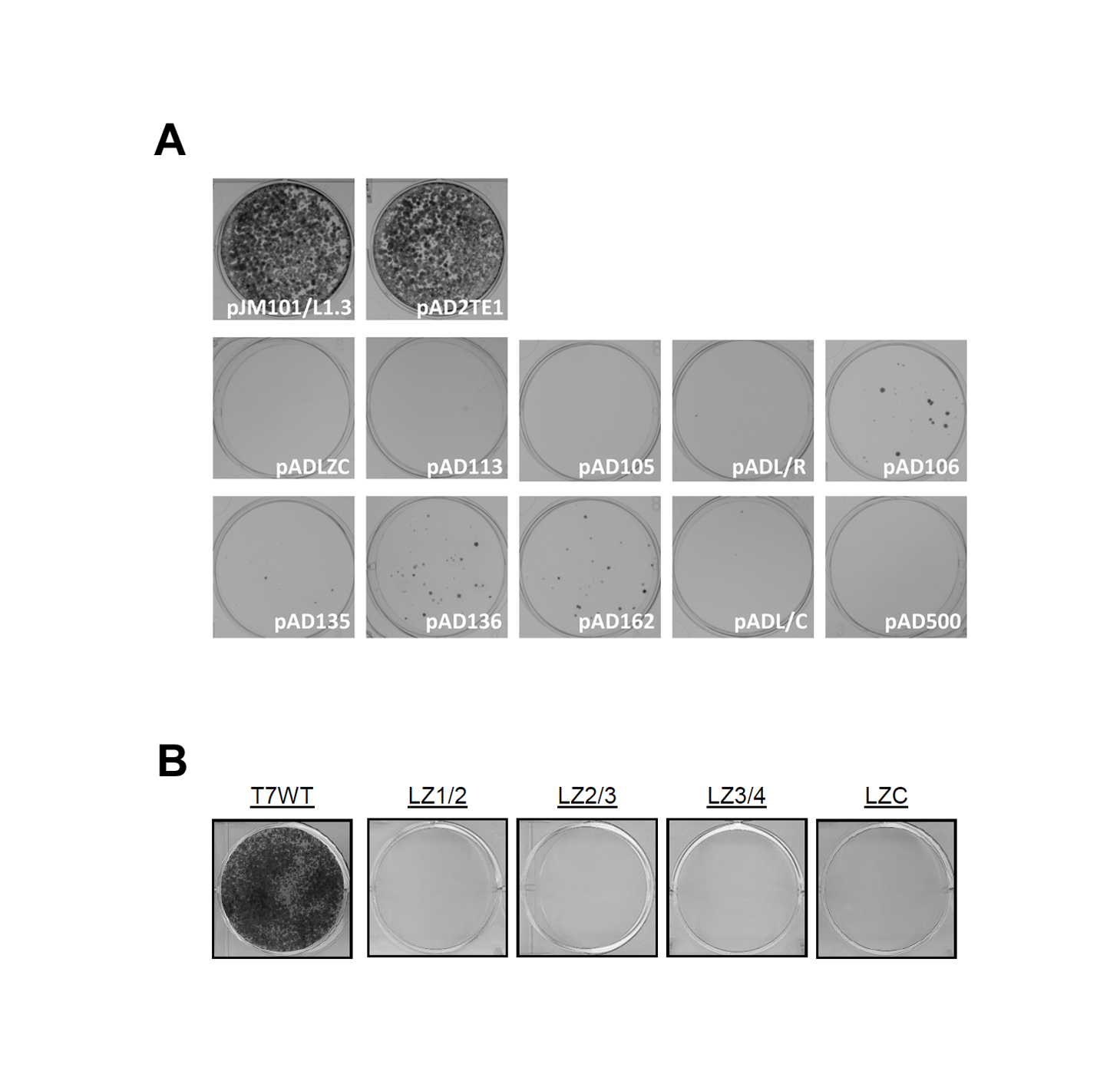

Supplement: Figure S1 — Retrotransposition assays with mutant L1 constructs. A. Retrotransposition assays with mutant L1 constructs: 2×10^4^ HeLa cells were transfected with the indicated constructs. pJM101/L1.3 and pAD2TE1 were used as positive controls. All of the pAD-based constructs contain the ORF1p T7 epitope tag and the ORF2p TAP-tag except for pAD500, which lacks ORF1. pAD135 is an RT mutant (D702A), and serves as a negative control. B. Retrotransposition assay with leucine zipper domain mutants: 2×10^5^ HeLa cells were transfected with the indicated pDK101-derived constructs. T7WT (pDK101) is a wild-type L1 (L1.3) that contains the T7 epitope tag on the carboxyl terminus of ORF1p. pDK101 was modified to create LZ1/2 (L93V, L100V), LZ2/3 (L100V, L107V), LZ3/4 (L107V, L114V), and LZC (L93V, L100V, L107V, L114V). Each of the mutations abolished L1 retrotransposition. (0.57 MB TIF) [file pgen.1001150.s001.tif]

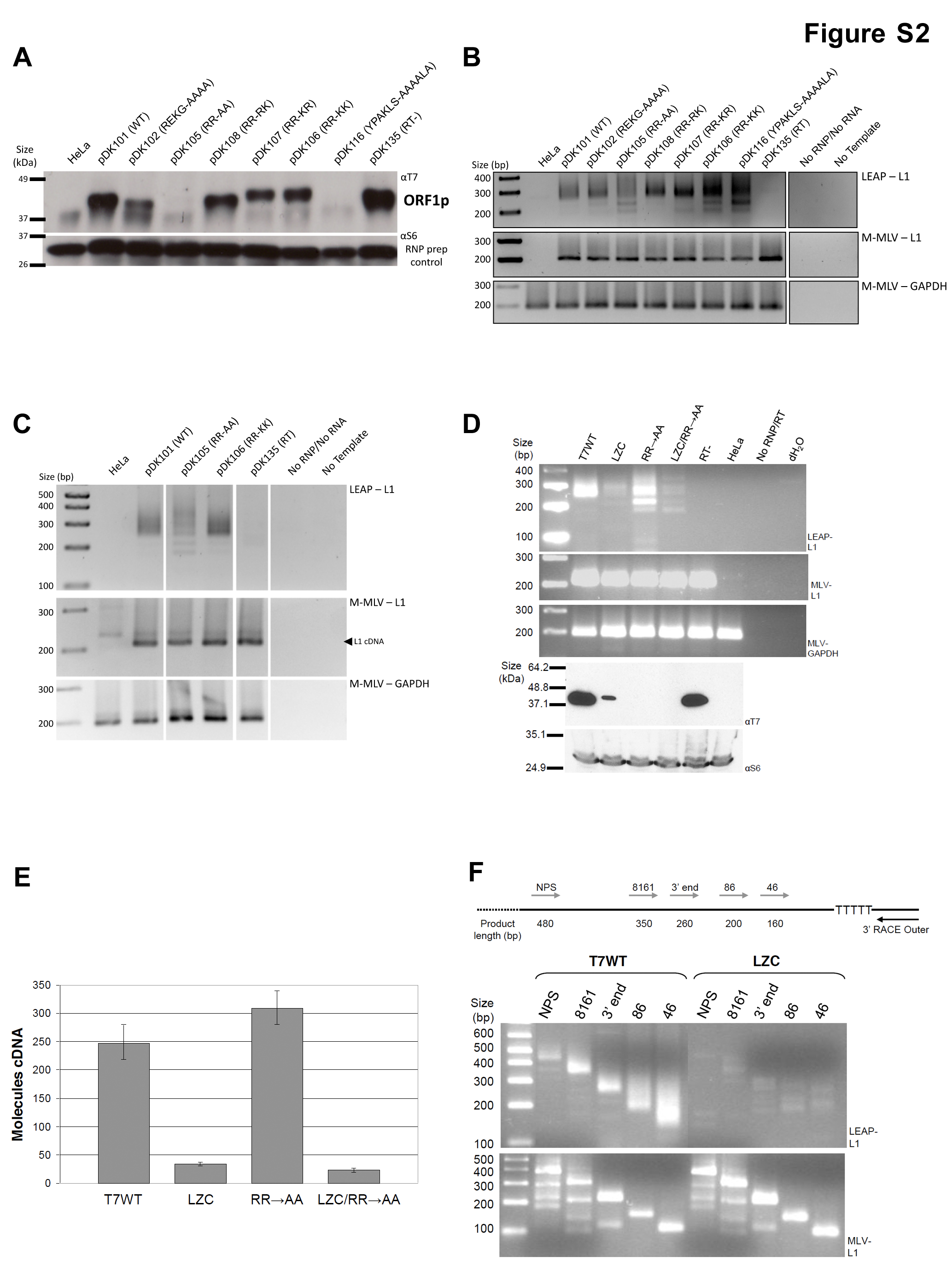

Supplement: Figure S2 — The effect of ORF1p mutations on LEAP activity. A. Results of western blot analyses: RNPs derived from wild-type (pDK101) and the indicated mutant constructs were subjected to western blot analyses with an anti-T7 antibody (αT7). An ∼40 kDa band indicative of epitope-tagged ORF1p is shown. Untransfected HeLa cells served as a negative control. The ribosomal S6 protein was detected using an anti-S6 (αS6) antibody (bottom panel; RNP prep control) and served as a loading control. Molecular weight markers (Invitrogen) are indicated at the left of the gel. B. Results of LEAP assays: Top panel: An aliquot of the above RNPs was used to measure LEAP activity. RNPs derived from wild-type (pDK101) generate strong LEAP products of ∼220–400 bp and served as a positive control. Untransfected HeLa cells and an RT mutant (pDK135; D702A) serve as negative controls. LEAP products generated in ORF1p mutant RNPs are shown. Reactions conducted without template (No Template) or without RNPs (No RNP/No RNA) were used as negative controls. Middle and bottom panels: RT-PCR with M-MLV RT and primers specific to either the transfected L1 constructs or GAPDH confirmed the presence of L1 RNA in RNPs and the integrity of the RNA isolation procedure. DNA size markers (Invitrogen) are indicated at the left of the gel. C. LEAP products derived from the ORF1p RNA binding mutant (RR261-262AA) and putative chaperone mutant (RR261-262KK): Representative LEAP products derived from wild-type (pDK101), an ORF1p RNA binding mutant (pDK105; RR261-262AA), and a putative ORF1p nucleic acid chaperone activity mutant (pDK106; RR261-262KK) are depicted at the top gel. The middle and bottom gels are RT-PCR reactions conducted with M-MLV RT and primers specific to L1 and GAPDH transcripts, respectively. The black arrow on the middle gel indicates the size of the specific L1 cDNA amplification products. Untransfected HeLa cells and a RT mutant (pDK135) served as negative controls. Additional negative controls incl [file pgen.1001150.s002.tif]

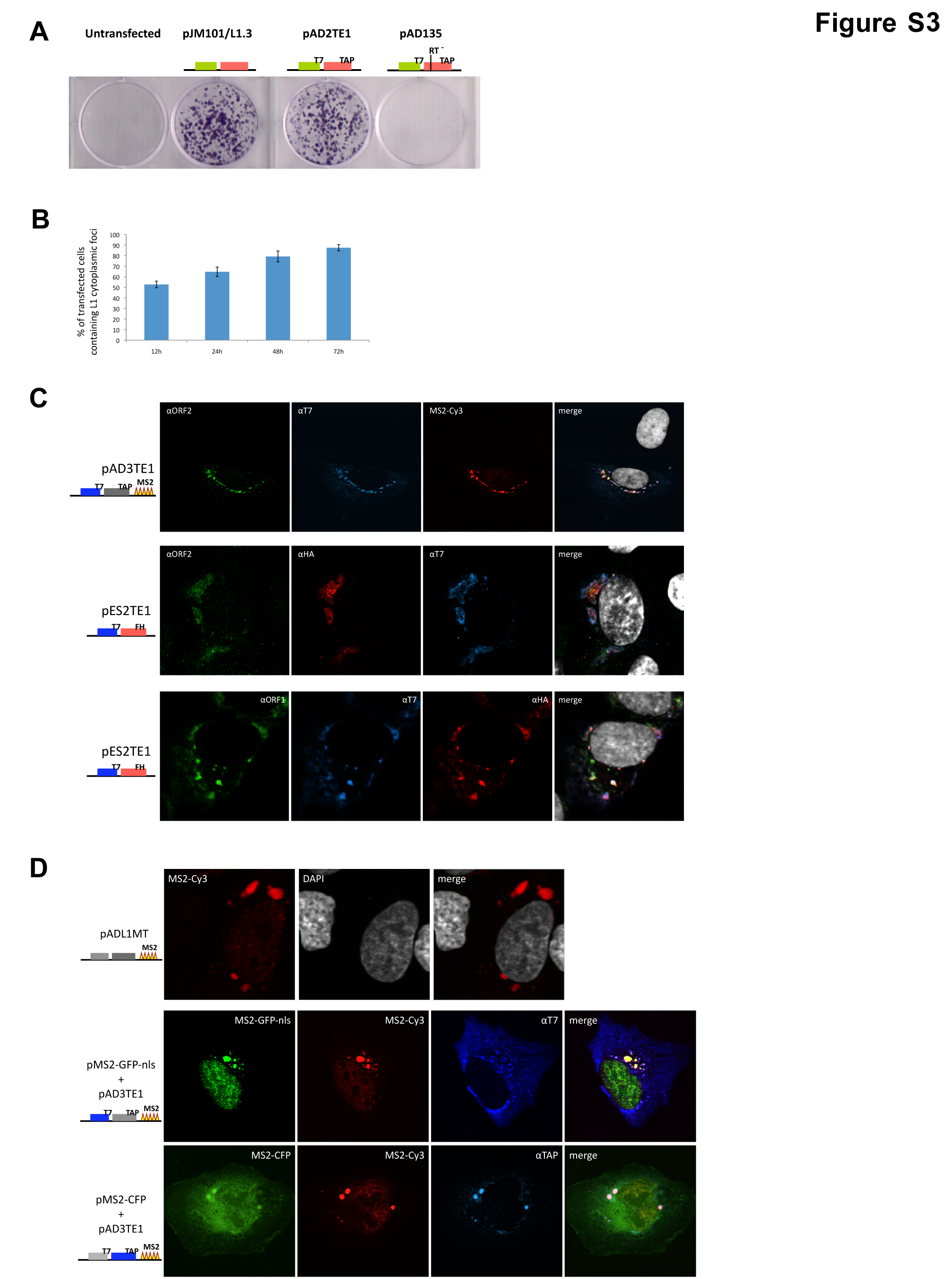

Supplement: Figure S3 — L1 retrotransposition and L1 cytoplasmic foci formation in U-2 OS cells. A. L1 retrotransposition assays: 2×10^4^ cells were transfected with the indicated L1 constructs. pJM101/L1.3 and pAD2TE1 were used as positive controls. Untransfected cells and pAD135 (RT mutant (D702A)) serve as negative controls. A cartoon of each L1 is shown above the tissue culture dishes. Green rectangle = ORF1; Red rectangle = ORF2. The relative positions of the T7 and TAP tags also are indicated. All constructs contain the mneoI retrotransposition indicator cassette. B. Time course analyses of L1 cytoplasmic foci formation: Cells were transfected with pAD2TE1. X-axis = time after transfection. Y-axis = percentage of transfected cells containing L1 cytoplasmic foci. For each time point, 100 transfected cells were analyzed for the presence of ORF1p and ORF2p in L1 cytoplasmic foci. Error bars = standard deviation (n = 3). C. Cytoplasmic localization of L1 proteins and RNA: Top panels: Cells were transfected with pAD3TE1. ORF2p was visualized with an anti-ORF2 antibody (αORF2, green). ORF1p was visualized with an anti-T7 antibody (αT7, blue). L1 RNA was visualized with an MS2-Cy3 FISH probe (red). A merged image is shown in the rightmost column; DAPI (grey) was used to stain nuclear DNA. Middle panels: Cells were transfected with pES2TE1. ORF2p was visualized with an anti-ORF2 antibody (αORF2, green) and an anti-HA antibody (αHA, red). ORF1p was visualized with an anti-T7 antibody (αT7, blue). A merged image is shown in the rightmost column; DAPI (grey) was used to stain nuclear DNA. Bottom panels: Cells were transfected with pES2TE1. ORF1p was visualized with an anti-ORF1 antibody (αORF1, green) and an anti-T7 antibody (αT7, blue). ORF2p was visualized with an anti-HA antibody (red). A merged image is shown in the rightmost column; DAPI (grey) was used to stain nuclear DNA. D. Localization of L1 RNA: Top panels: Cells were transfected with pADL1MT. L1 RNA was visualized with an MS2-Cy3 FISH [file pgen.1001150.s003.tif]
